# Natural Products in Diabetes Mellitus: A Narrative Review of Mechanistic Insights into Metabolic Regulation and Therapeutic Potential

**DOI:** 10.3390/nu18203318

**Published:** 2026-10-10

**Authors:** Hemalata Deshmukh, Ganesh J. Kshirsagar, Lauren Gollahon

**Affiliations:** Department of Biological Sciences, Texas Tech University, 2500 Broadway, Lubbock, TX 79409, USA; hemalata.deshmukh@ttu.edu (H.D.); ganesh.kshirsagar@ttu.edu (G.J.K.)

**Keywords:** natural products, diabetes mellitus, oxidative stress, inflammation, insulin resistance, mitochondrial dysfunction, metabolic regulation

## Abstract

Diabetes mellitus is a complex metabolic disease characterized by persistent hyperglycemia resulting from impaired insulin secretion, insulin action, or both. In addition to disturbances in glucose metabolism, the disease is accompanied by abnormalities in mitochondrial dysfunction, oxidative stress, lipid and protein metabolism, and chronic inflammation that contribute to progressive tissue injury and the development of vascular complications. Although current pharmacological therapies effectively improve glycemic control, many patients continue to experience disease progression and diabetes-associated complications, highlighting the need for complementary therapeutic strategies that target multiple pathogenic mechanisms. Growing evidence suggests that naturally occurring bioactive compounds may provide such multitarget benefits. Plant-derived polyphenols, medicinal herbs, culinary spices, and functional foods have demonstrated antioxidant, anti-inflammatory, hypoglycemic, hypolipidemic, and insulin-sensitizing properties in experimental and clinical studies. Compounds including curcumin, honey, garlic, fenugreek, cumin, shilajit, and guduchi have been reported to influence several molecular pathways involved in diabetes pathogenesis, including NF-κB-mediated inflammatory signaling, oxidative and endoplasmic reticulum stress, PPARγ activation, advanced glycation end-product formation, and pancreatic β-cell preservation. This review critically examines current evidence regarding the role of natural products in diabetes management, integrating findings from cell-based studies, animal models, and human clinical investigations. Particular emphasis is placed on the interplay between oxidative stress, inflammation, mitochondrial dysfunction, and metabolic regulation, as these interconnected processes represent key drivers of disease progression. In addition to summarizing established mechanisms, the review discusses current limitations, translational challenges, and future research priorities. A deeper understanding of how natural products modulate these pathways may facilitate the development of evidence-based adjunctive strategies to improve long-term metabolic health and reduce the burden of diabetic complications. This narrative review critically synthesizes published peer-reviewed mechanistic, preclinical, and clinical evidence regarding selected natural products and functional foods relevant to diabetes mellitus.

## 1. Introduction

Diabetes mellitus is one of the most prevalent non-communicable chronic metabolic diseases worldwide and remains a major public health challenge [1]. The global prevalence of both type 1 diabetes (T1DM) and type 2 diabetes (T2DM) has increased steadily over recent decades, largely because of population aging, urbanization, reduced physical activity, unhealthy dietary habits, and the growing prevalence of obesity [2]. Among these conditions, T2DM accounts for nearly 90–95% of all diagnosed cases and is characterized by progressive insulin resistance accompanied by inadequate pancreatic β-cell compensation [3]. In contrast, T1DM results primarily from autoimmune destruction of insulin-producing β-cells [4]. Although their underlying etiologies differ, both forms ultimately share common metabolic disturbances that include persistent hyperglycemia, dysregulated lipid metabolism, oxidative stress, mitochondrial dysfunction, and chronic inflammation [5]. Collectively, these interconnected metabolic abnormalities contribute not only to impaired glucose homeostasis but also to the development of microvascular and macrovascular complications that remain the principal causes of diabetes-associated morbidity and mortality [6]. Although this review considers mechanisms relevant to both T1DM and T2DM, the majority of experimental and clinical evidence concerning the natural products discussed relates to T2DM, insulin resistance, obesity-associated metabolic dysfunction, and progressive β-cell impairment. T1DM is fundamentally characterized by autoimmune destruction of pancreatic β-cells, whereas T2DM is driven predominantly by insulin resistance, compensatory hyperinsulinemia, and progressive β-cell dysfunction. Accordingly, evidence obtained from metabolic models of T2DM should not be directly extrapolated to autoimmune T1DM [3,4,5,7,8].

Persistent hyperglycemia is not merely a biochemical hallmark of diabetes but also a major contributor to disease progression and long-term complications [9,10]. Elevated glucose concentrations promote the formation of advanced glycation end products (AGEs), which alter protein function, disrupt cellular homeostasis, and activate inflammatory signaling pathways [11,12]. Interactions between AGEs and their receptors (RAGE) further amplify oxidative stress and inflammatory responses through activation of nuclear factor-κB (NF-κB), thereby contributing to vascular dysfunction and progressive tissue injury [12,13].

Concurrently, excessive glucose and fatty acid availability increases mitochondrial oxidative metabolism, leading to overproduction of reactive oxygen species (ROS) [9,10]. The resulting oxidative stress impairs insulin signaling, damages pancreatic β-cells, and accelerates the development of diabetic nephropathy, retinopathy, neuropathy, and cardiovascular disease [9,10,12]. These interconnected mechanisms emphasize that diabetes is driven by a network of metabolic abnormalities rather than hyperglycemia alone [9,10]. The gut microbiota has also emerged as an important regulator of metabolic homeostasis. Alterations in microbial composition and function may influence intestinal barrier integrity, short-chain fatty acid production, bile-acid metabolism, inflammatory signaling, endotoxin exposure, and insulin sensitivity [14]. Dietary phytochemicals and functional foods may interact bidirectionally with the gut microbiota, because microbial metabolism can generate bioactive metabolites while natural products can alter microbial composition and activity. These interactions may therefore contribute to some of the metabolic effects attributed to dietary natural products, although causal relationships remain incompletely defined [14,15].

Over the past several decades, substantial progress has been made in the pharmacological management of diabetes. Current therapeutic options include insulin, metformin, sulfonylureas, thiazolidinediones, incretin-based therapies, and sodium-glucose cotransporter-2 (SGLT2) inhibitors—all of which have significantly improved glycemic control and reduced the risk of certain complications [16]. Nevertheless, many patients fail to achieve sustained metabolic control, while adverse effects, treatment costs, and progressive loss of therapeutic efficacy remain important clinical challenges [16]. Furthermore, most available drugs primarily target glucose regulation and only partially address the oxidative, inflammatory, and mitochondrial abnormalities that contribute to disease progression [10,16].

These limitations have renewed interest in natural products as complementary approaches for diabetes management. Medicinal plants, dietary phytochemicals, culinary spices, and traditional herbal formulations have long been used in systems of medicine such as Ayurveda and Traditional Chinese Medicine for the treatment of metabolic disorders [17]. Unlike single-target pharmacological agents, many naturally occurring compounds contain multiple bioactive constituents capable of influencing several molecular pathways simultaneously [18]. Such multitarget activity is particularly attractive in diabetes, where disturbances in metabolism, inflammation, oxidative stress, and cellular signaling occur concurrently and interact throughout disease progression [10,18].

Advances in molecular biology, metabolomics, and redox biology have considerably expanded our understanding of how natural compounds influence metabolic homeostasis. Polyphenols such as curcumin, for example, regulate transcription factors, protein kinases, and nuclear receptors involved in insulin signaling, mitochondrial function, oxidative stress responses, and inflammatory pathways [18,19,20]. Likewise, functional foods including honey and commonly consumed spices have demonstrated biological activities that extend beyond their nutritional value by influencing glucose metabolism, lipid handling, antioxidant defenses, and immune responses [21,22,23]. These mechanistic insights provide a stronger scientific basis for evaluating natural products as adjuvants to conventional diabetes therapy rather than simply alternative remedies.

The objective of this review is to critically evaluate current evidence regarding the role of natural products in the prevention and management of diabetes mellitus, with particular emphasis on the molecular and biochemical mechanisms underlying their therapeutic effects. Findings from in vitro experiments, animal studies, and clinical investigations are integrated to examine how these compounds influence oxidative stress, inflammatory signaling, mitochondrial function, insulin sensitivity, and glucose metabolism. In addition to summarizing the available evidence, this review discusses existing knowledge gaps, translational challenges, and future research priorities that should be addressed before these natural products can be more effectively incorporated into evidence-based strategies for diabetes management.

## 2. Literature Search and Review Approach

This article was prepared as a narrative review to provide a broad and critical synthesis of published peer-reviewed literature concerning natural products and functional foods in diabetes mellitus. Relevant literature was identified through searches of Pubmed, supplemented by examination of reference lists of relevant original studies and review articles. Search terms included combinations of “diabetes mellitus, “type 1 diabetes,” “type 2 diabetes,” “insulin resistance,” “natural products,” “functional foods,” “curcumin,” “honey,” “garlic,” “fenugreek,” “cumin,” “coriander,” “ginger,” “cinnamon,” “shilajit,” “guduchi,” “oxidative stress,” “inflammation,” “mitochondrial dysfunction,” and related terms. Priority was given to peer-reviewed mechanistic studies, relevant animal studies, controlled clinical investigations, randomized clinical trials, systematic reviews, and meta-analyses where available. The present article was not designed as a systematic review or meta-analysis; therefore, formal PRISMA-based screening, risk-of-bias assessment, and quantitative pooling of effect estimates were not performed.

## 3. Pathophysiology of Diabetes Mellitus: Metabolic, Redox, and Inflammatory Dysregulation

Diabetes mellitus is a multifaceted metabolic disorder that encompasses far more than impaired glucose regulation [10,24]. Although chronic hyperglycemia defines the disease clinically, its progression is driven by a complex interaction between insulin resistance, pancreatic β-cell dysfunction, mitochondrial abnormalities, oxidative stress, chronic inflammation, and altered lipid metabolism [9,10,24]. These processes are closely interconnected and reinforce one another, creating a continuous cycle that contributes to progressive metabolic dysfunction and the development of microvascular and macrovascular complications [9,12,24]. Understanding these interconnected mechanisms provides the biological basis for therapeutic strategies capable of targeting multiple pathways simultaneously [18,24]. Chemically induced diabetes models using streptozotocin or alloxan are useful for investigating β-cell injury, insulin deficiency, hyperglycemia, and oxidative stress; however, these models do not reproduce the autoimmune pathogenesis of human T1DM [25,26]. They should therefore be described as chemically induced β-cell injury or insulin-deficient models rather than direct models of autoimmune T1DM. In contrast, diet-induced obesity and insulin-resistance models reproduce selected metabolic features of T2DM [26].

### 3.1. Insulin Resistance and Impaired Glucose Homeostasis

Insulin resistance is a central pathophysiological feature of type 2 diabetes mellitus and can develop years before the onset of clinically overt hyperglycemia [7]. Under physiological conditions, insulin promotes glucose uptake in skeletal muscle and adipose tissue while suppressing hepatic glucose production [7]. These effects rely on coordinated activation of the insulin receptor and downstream signaling molecules, including insulin receptor substrate (IRS) proteins, phosphoinositide 3-kinase (PI3K), and protein kinase B (Akt). Disruption of this signaling cascade diminishes GLUT4 translocation to the plasma membrane, resulting in impaired glucose uptake and reduced glycogen synthesis [7,9].

Insulin resistance also alters hepatic metabolism. Rather than suppressing glucose production, the insulin-resistant liver continues to generate glucose through gluconeogenesis, further aggravating hyperglycemia [7]. Initially, pancreatic β-cells compensate by increasing insulin secretion, leading to hyperinsulinemia. However, sustained secretory demand ultimately overwhelms β-cell function, causing impaired insulin synthesis, defective proinsulin processing, and gradual β-cell loss [8]. The transition from compensated insulin resistance to β-cell failure represents a critical step in the progression toward overt diabetes [7,8]. Glucose homeostasis is regulated by a coordinated endocrine network rather than insulin alone. Glucagon promotes hepatic glucose production, whereas incretin hormones such as glucagon-like peptide-1 and glucose-dependent insulinotropic polypeptide regulate meal-stimulated insulin secretion and glucagon release [27]. Adiponectin and leptin influence insulin sensitivity, lipid metabolism, energy balance, and inflammatory signaling, while cortisol and catecholamines act as counter-regulatory hormones that can increase hepatic glucose production [28]. Dysregulation of these hormonal pathways contributes to metabolic imbalance in diabetes and interacts with oxidative and inflammatory mechanisms [29,30].

### 3.2. Lipotoxicity and Dysregulated Lipid Metabolism

Abnormal lipid metabolism plays a central role in both the onset and progression of diabetes. Elevated circulating free fatty acids, commonly observed in obesity and metabolic syndrome, accumulate within insulin-responsive tissues where they interfere with intracellular insulin signaling [31]. Bioactive lipid intermediates, including ceramides and diacylglycerols, activate stress-related kinases that inhibit IRS function through serine phosphorylation, thereby weakening insulin responsiveness [31,32].

The consequences of lipotoxicity extend beyond insulin resistance. Diabetic dyslipidemia, characterized by elevated triglycerides and low-density lipoprotein cholesterol together with reduced high-density lipoprotein cholesterol, substantially increases cardiovascular risk [33]. Pancreatic β-cells are particularly susceptible because of their limited capacity to store excess lipids and relatively weak antioxidant defenses. Accumulation of toxic lipid species within β-cells enhances oxidative stress, promotes mitochondrial dysfunction, and accelerates apoptotic cell death, ultimately compromising insulin secretion [34].

### 3.3. Mitochondrial Dysfunction in Diabetes

Mitochondria occupy a preeminent role in the regulation of cellular energy metabolism and are therefore critical determinants of metabolic health. In diabetes, impaired mitochondrial function reduces oxidative phosphorylation efficiency, limiting ATP production while simultaneously increasing the generation of reactive oxygen species (ROS) [9,35]. In metabolically active tissues such as skeletal muscle and liver, reduced mitochondrial oxidative capacity impairs fatty acid oxidation, thereby promoting intracellular lipid accumulation and reinforcing insulin resistance [35].

Pancreatic β-cells are especially dependent on mitochondrial metabolism because insulin secretion is directly coupled with ATP production [36]. Any disruption of mitochondrial bioenergetics compromises glucose-stimulated insulin release. Moreover, excessive mitochondrial ROS damages mitochondrial DNA, proteins, and membrane lipids, creating a vicious cycle in which oxidative injury further impairs mitochondrial function and cellular energy production [9,36,37]. This progressive decline contributes significantly to β-cell dysfunction during diabetes [36,37].

### 3.4. Oxidative Stress as a Central Pathogenic Mechanism

Oxidative stress has emerged as a fundamental mechanism linking metabolic dysfunction to diabetic complications [9,10]. Chronic hyperglycemia promotes excessive ROS generation through several pathways, including glucose auto-oxidation, activation of the polyol pathway, increased formation of advanced glycation end products, and overload of the mitochondrial electron transport chain [9,10]. Elevated circulating fatty acids further amplify oxidative stress by increasing mitochondrial substrate availability and electron leakage [10].

Excessive ROS disrupts normal cellular function through oxidative modification of proteins, membrane lipids, and nucleic acids [10,38]. In addition to causing structural damage, ROS interfere directly with insulin signaling by altering key signaling proteins involved in glucose transport and metabolism. The resulting oxidative environment contributes to endothelial dysfunction, accelerates vascular injury, and promotes the progression of nephropathy, retinopathy, and peripheral neuropathy [10,38]. Because pancreatic β-cells express relatively low levels of endogenous antioxidant enzymes, they are particularly vulnerable to oxidative injury, making oxidative stress a major contributor to the progressive decline in insulin secretory capacity [39].

### 3.5. Chronic Inflammation and Stress-Activated Signaling Pathways

Accumulating evidence indicates that chronic low-grade inflammation is closely linked to insulin resistance and metabolic dysfunction. Expansion of adipose tissue in obesity promotes infiltration of macrophages and other immune cells, resulting in increased production of pro-inflammatory mediators such as tumor necrosis factor-α (TNF-α), interleukin-6 (IL-6), and monocyte chemoattractant protein-1 (MCP-1) [40,41]. These cytokines activate intracellular signaling pathways that interfere with insulin receptor function and impair metabolic regulation [13,32,41].

Among these pathways, NF-κB, c-Jun N-terminal kinase (JNK), and inhibitor of κB kinase (IKK) are particularly important because they integrate inflammatory and metabolic signaling. Activation of these pathways suppresses insulin signaling while simultaneously promoting expression of additional inflammatory mediators, thereby establishing a positive feedback loop that sustains tissue inflammation and insulin resistance [13,32,41]. Nutrient excess also induces endoplasmic reticulum stress, which further amplifies inflammatory signaling and contributes to β-cell dysfunction and insulin resistance [42]. Together, oxidative stress, inflammation, and endoplasmic reticulum stress form an interconnected network that drives disease progression [13,32,42].

### 3.6. Integrated Perspective and Therapeutic Implications

Rather than arising from a single biochemical abnormality, diabetes mellitus develops through the convergence of multiple pathological mechanisms. Hyperglycemia, insulin resistance, lipotoxicity, mitochondrial dysfunction, oxidative stress, and chronic inflammation interact through interconnected pathways, each capable of exacerbating the others and accelerating metabolic deterioration [9,10]. This systems-level complexity helps explain why strategies focused predominantly on glycemic control may not completely prevent the development or progression of diabetes-associated complications [10].

These mechanistic insights provide a rationale for investigating natural products as potential adjuncts to conventional diabetes management. Many bioactive phytochemicals exhibit antioxidant, anti-inflammatory, insulin-sensitizing, and glucose- and lipid-modulating activities and can influence multiple molecular pathways involved in diabetes pathophysiology [43,44]. Their multitarget actions may therefore complement conventional pharmacological approaches by modulating interconnected metabolic, oxidative, and inflammatory pathways rather than glycemia alone [44]. The following sections examine how selected natural products modulate these pathways and critically evaluate the experimental and clinical evidence supporting their potential role as adjunctive strategies in diabetes management.

## 4. Oxidative Stress and Inflammatory Signaling in Diabetes Mellitus

Oxidative stress and chronic inflammation are now recognized as central mechanisms underlying the development and progression of diabetes mellitus [9,10,24]. Rather than acting as independent pathological processes, they interact through multiple feedback loops that impair insulin signaling, damage pancreatic β-cells, and promote vascular complications [10,24]. Persistent metabolic overload increases reactive oxygen species (ROS) production while simultaneously weakening endogenous antioxidant defenses, creating a pro-oxidant environment that sustains inflammation and accelerates tissue injury [9,10]. Understanding this reciprocal relationship is essential for identifying therapeutic strategies that extend beyond glucose lowering and target the molecular drivers of diabetic complications [24].

### 4.1. Sources of Reactive Oxygen Species in Hyperglycemic States

Under physiological conditions, ROS are continuously generated as by-products of normal cellular metabolism and participate in intracellular signaling [10,24]. In diabetes, however, chronic hyperglycemia and elevated circulating fatty acids markedly increase ROS production, overwhelming antioxidant defense systems [9,10]. Mitochondria represent the principal source of oxidative stress in metabolically active tissues. Excess nutrient availability increases electron flow through the mitochondrial electron transport chain, promoting electron leakage and the formation of superoxide radicals. When antioxidant capacity becomes insufficient, these radicals accumulate and initiate oxidative damage to cellular macromolecules [10].

**Figure 1:**
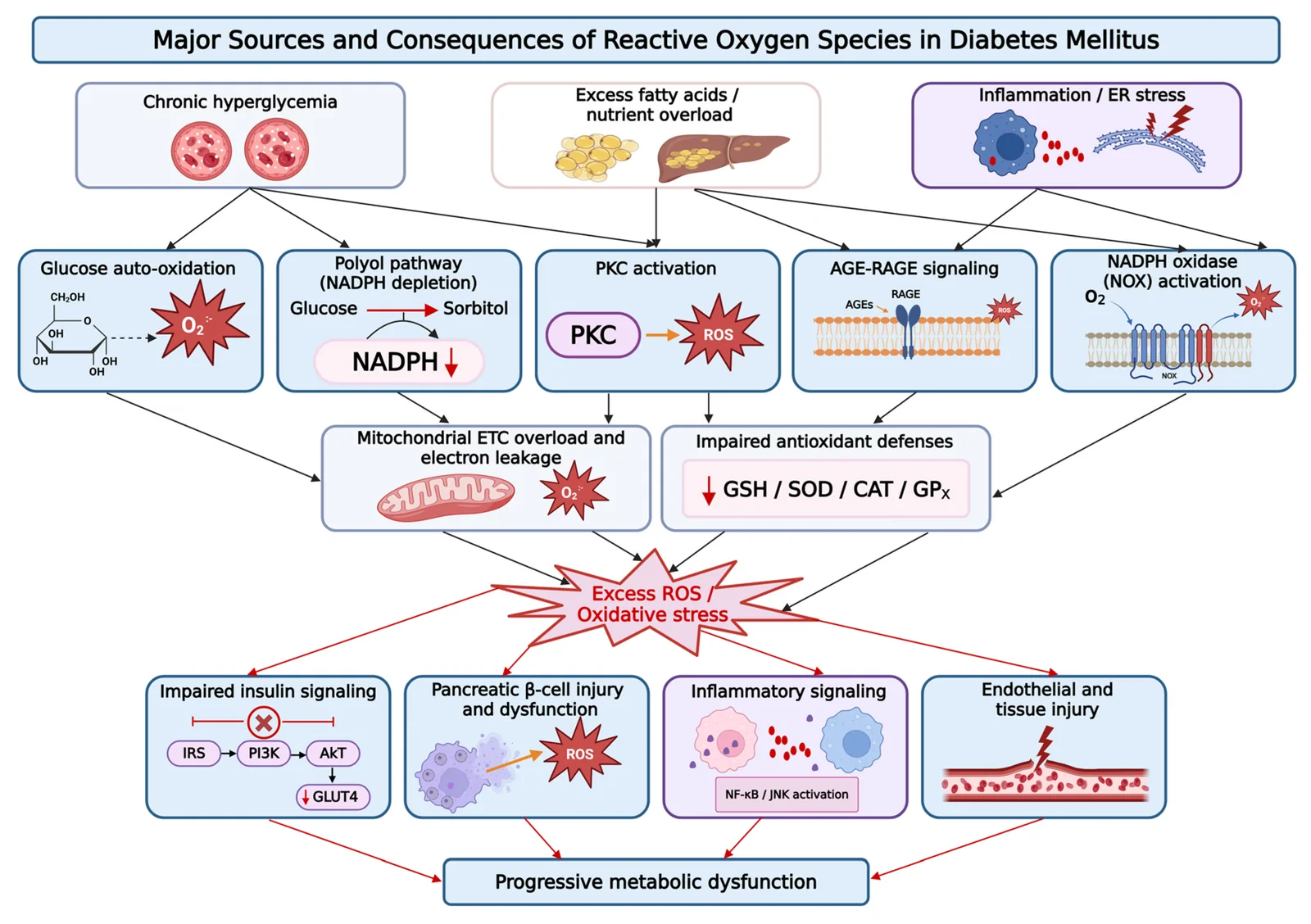
Major sources and downstream consequences of reactive oxygen species in diabetes mellitus. Chronic hyperglycemia, excess fatty-acid availability, inflammatory signaling, and endoplasmic reticulum stress promote excessive reactive oxygen species (ROS) generation through several interconnected pathways, including mitochondrial electron-transport-chain overload and electron leakage, glucose auto-oxidation, activation of the polyol pathway and protein kinase C, AGE–RAGE signaling, and NADPH oxidase activation. At the same time, impairment of endogenous antioxidant defenses, including reduced glutathione availability and decreased activity of antioxidant enzymes such as superoxide dismutase, catalase, and glutathione peroxidase, further contributes to oxidative stress. Excess ROS can disrupt IRS–PI3K–Akt signaling and GLUT4-mediated glucose transport, promote pancreatic β-cell injury, activate inflammatory pathways such as NF-κB and JNK, and contribute to endothelial and tissue damage.

Mitochondrial dysfunction is not the sole contributor to oxidative stress. Additional ROS arise from glucose auto-oxidation, activation of the polyol pathway, protein kinase C signaling, and excessive formation of advanced glycation end products (AGEs) and activation of NADPH oxidases (NOXs) [9,10]. Increased flux through the polyol pathway consumes NADPH, limiting glutathione regeneration and weakening antioxidant defenses. Meanwhile, interactions between AGEs and their receptor (RAGE) amplify oxidative stress by stimulating both inflammatory and pro-oxidant signaling cascades [10,45]. These multiple sources collectively create a sustained oxidative environment characteristic of diabetic tissues shown in Figure 1.

### 4.2. Antioxidant Defense Systems and Their Impairment in Diabetes

Cellular resistance to oxidative injury depends on a coordinated antioxidant network comprising enzymatic components, including superoxide dismutase (SOD), catalase, and glutathione peroxidase (GPx), together with non-enzymatic antioxidants such as glutathione, vitamins, and naturally occurring polyphenols [9,10]. In diabetes, persistent oxidative stress can disrupt this protective network through alterations in antioxidant enzyme activity and depletion of intracellular glutathione, thereby reducing cellular capacity to maintain redox homeostasis and neutralize excessive ROS [10,39].

Pancreatic β-cells are particularly sensitive to disturbances in redox homeostasis because their antioxidant capacity is relatively modest compared with many other cell types [39]. Consequently, persistent excessive ROS can impair mitochondrial function and ATP generation, disrupt glucose-stimulated insulin secretion, and activate cellular stress and apoptotic pathways [10,39]. Progressive oxidative injury can therefore contribute to β-cell dysfunction and declining insulin secretory capacity in both type 1 and type 2 diabetes, although the initiating mechanisms and relative contribution of oxidative stress differ between the two forms of the disease [10].

### 4.3. Oxidative Stress—Mediated Disruption of Insulin Signaling

In addition to causing structural cellular damage, ROS profoundly influence intracellular signaling pathways involved in glucose metabolism [9,10]. Oxidative stress activates stress-responsive kinases such as c-Jun N-terminal kinase (JNK) and p38 mitogen-activated protein kinase, which phosphorylate insulin receptor substrate proteins at inhibitory serine residues [9,13,32]. This modification suppresses PI3K-Akt signaling, reducing glucose uptake, glycogen synthesis, and overall insulin responsiveness [9,10].

ROS also promote oxidative modification of membrane lipids and signaling proteins required for insulin receptor activation and GLUT4 trafficking [9,10]. These alterations further impair glucose transport in skeletal muscle and adipose tissue, reinforcing systemic insulin resistance [9,10,32]. Consequently, oxidative stress is no longer viewed solely as a consequence of diabetes but also as an active participant in disease initiation and progression [9,10].

### 4.4. Inflammatory Pathways Linking Metabolism and Immune Activation

Chronic low-grade inflammation has become a defining feature of obesity-associated insulin resistance and type 2 diabetes. Expansion and dysfunction of adipose tissue promote the recruitment and activation of macrophages and other immune cells, leading to increased production of pro-inflammatory cytokines, including tumor necrosis factor-α (TNF-α), interleukin-6 (IL-6), and interleukin-1β (IL-1β) [40,46]. These inflammatory mediators disrupt metabolic homeostasis by activating stress-sensitive signaling pathways that interfere with insulin signaling in insulin-responsive tissues [13,32,46].

At the molecular level, inflammatory responses are mediated by signaling pathways involving nuclear factor-κB (NF-κB), inhibitor of κB kinase (IKK), and c-Jun N-terminal kinase (JNK) [13,32]. Activation of the IKK/NF-κB axis promotes the transcription of numerous pro-inflammatory cytokines, chemokines, and adhesion molecules while contributing to impaired insulin signaling [13]. JNK activation further disrupts insulin signaling, in part through inhibitory serine phosphorylation of insulin receptor substrate (IRS) proteins, thereby strengthening the mechanistic link between immune activation and metabolic dysfunction [32,46].

### 4.5. Crosstalk Between Oxidative Stress and Inflammation

Oxidative stress and inflammation form an interconnected pathogenic network rather than separate biological processes. ROS activate redox-sensitive transcription factors including NF-κB and activator protein-1 (AP-1), leading to increased expression of inflammatory mediators [9,10,13,24]. In turn, inflammatory cytokines stimulate mitochondrial dysfunction and NADPH oxidase activity, generating additional ROS and perpetuating oxidative injury [10,24]. This reciprocal amplification establishes a self-sustaining cycle that contributes to progressive insulin resistance and tissue damage [9,24].

Endoplasmic reticulum stress provides another important mechanistic link between metabolism and inflammation. Nutrient overload disrupts protein folding, activating the unfolded protein response [42]. Persistent activation of this pathway enhances inflammatory signaling, promotes oxidative stress, and further compromises insulin signaling and β-cell survival. These overlapping pathways illustrate the highly integrated nature of metabolic dysfunction in diabetes [10,42].

### 4.6. Implications for Therapeutic Targeting

Recognition of oxidative stress and inflammation as fundamental drivers of diabetes has broadened the therapeutic focus beyond glycemic control alone [9,10]. Although conventional antidiabetic drugs effectively lower blood glucose concentrations, their influence on oxidative injury and inflammatory signaling is often limited. This has prompted growing interest in adjunctive interventions that can restore redox balance while simultaneously suppressing chronic inflammation [44].

**Figure 2:**
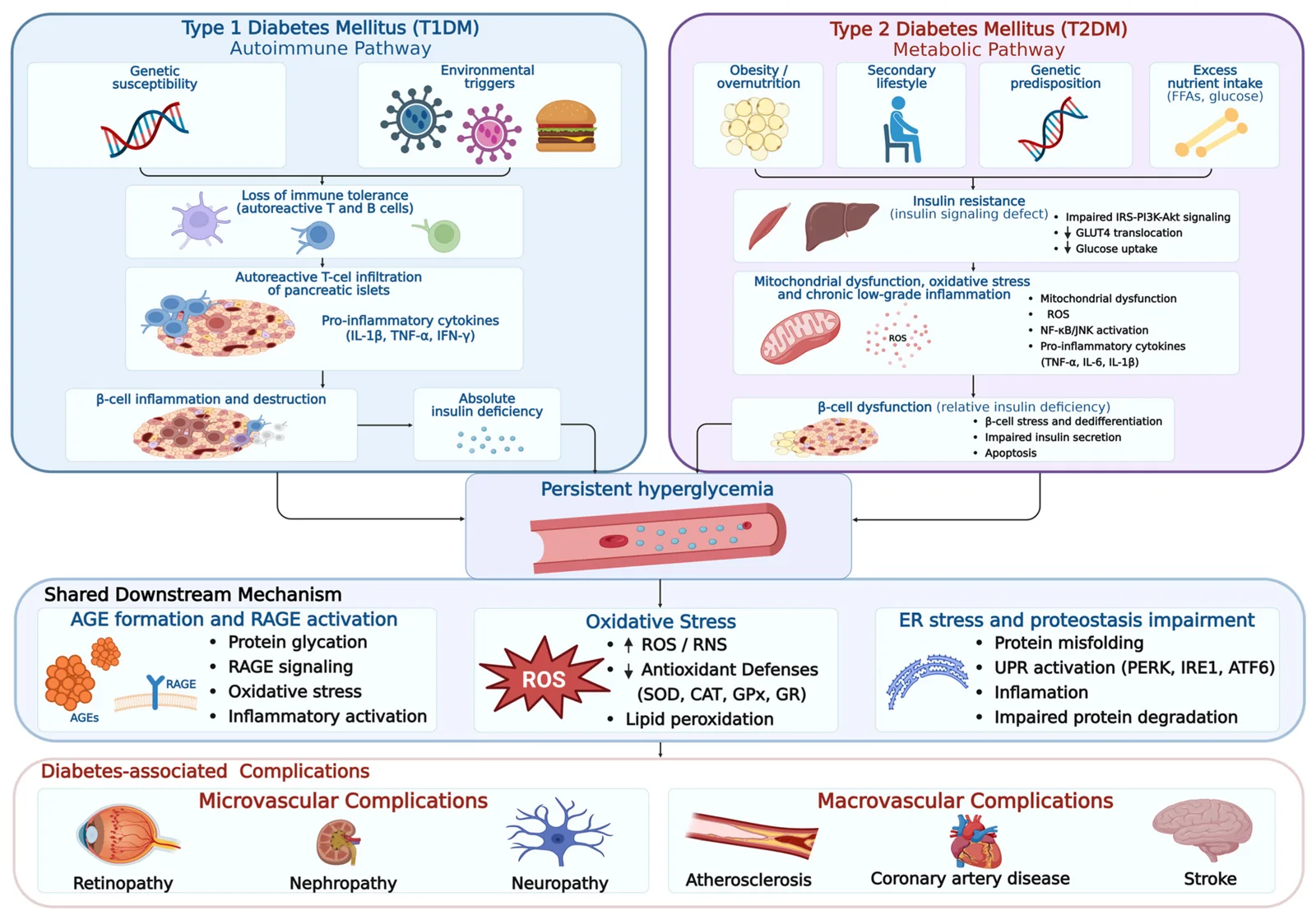
Integrated pathogenesis of T1DM and T2DM. T1DM is initiated by autoimmune β-cell destruction leading to absolute insulin deficiency, whereas T2DM is primarily driven by insulin resistance and progressive β-cell dysfunction. Both pathways converge on hyperglycemia, oxidative and ER stress, AGE–RAGE signaling, and inflammation, contributing to microvascular and macrovascular diabetic complications.

Many naturally occurring bioactive compounds possess antioxidant and anti-inflammatory properties that influence multiple molecular targets simultaneously [18,19,44]. Polyphenols, flavonoids, and other phytochemicals have been shown to regulate NF-κB signaling, inhibit stress-activated kinases, enhance endogenous antioxidant defenses, and preserve mitochondrial function. Through these coordinated actions, natural products may improve insulin sensitivity, protect pancreatic β-cells, and reduce the progression of diabetes-related complications [18,44]. The following sections discuss selected natural compounds whose mechanisms of action have been investigated extensively in experimental and clinical studies. The natural products discussed in this review were selected as representative examples based on their dietary or traditional relevance, availability of mechanistic and/or clinical evidence, and their ability to illustrate distinct pathways involved in glucose metabolism, oxidative stress, inflammation, insulin sensitivity, mitochondrial function, or β-cell biology. The selection is illustrative rather than exhaustive and should not be interpreted as a ranking or endorsement of the included products. The interconnected pathogenic mechanisms underlying diabetes mellitus and its associated complications as well as the links to oxidative stress, inflammatory signaling, and metabolic dysfunction in diabetes are summarized in Figure 2.

## 5. Natural Products as Multitarget Antidiabetic Agents

**Figure 3:**
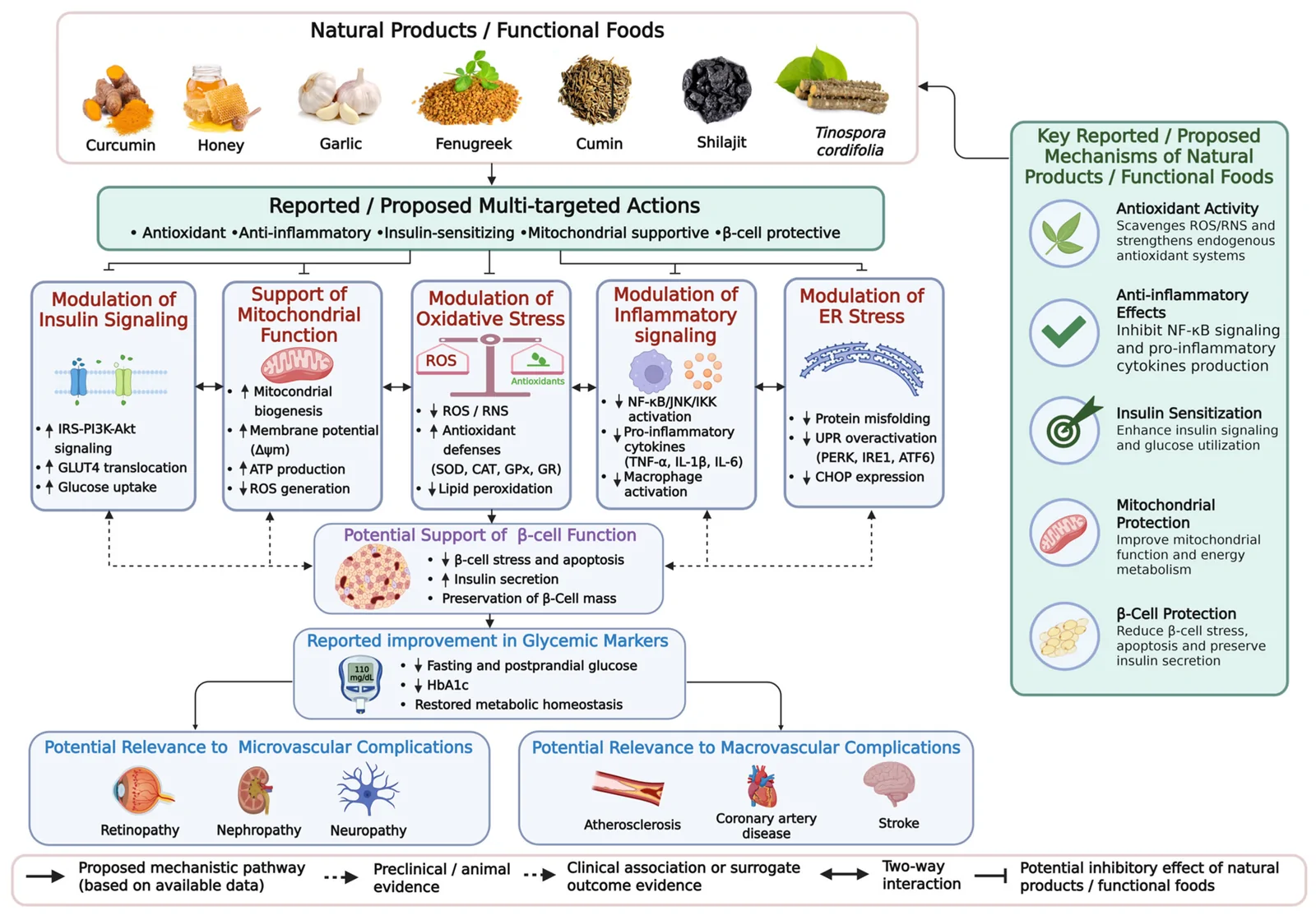
Proposed mechanisms by which natural products and functional foods may influence metabolic dysfunction and diabetes-associated complications. Curcumin has been reported to modulate insulin signaling, oxidative stress, inflammatory pathways, mitochondrial function, and ER stress [19,20,47,48,49,50,51,52]. Honey has been associated with antioxidant, anti-inflammatory, and mitochondrial protective effects [21,53,54,55,56,57,58]. Garlic may improve insulin signaling and antioxidant defenses and modulate inflammatory and ER-stress pathways [59,60,61,62,63,64]. Fenugreek has been linked to improved insulin sensitivity, attenuation of oxidative stress and inflammation, and β-cell protection [65,66,67,68,69,70,71]. Cumin has been reported to influence glucose uptake, AMPK/SIRT1-associated signaling, mitochondrial function, and proteostasis [19,20,47,48,50,52,72]. Shilajit has antioxidant, insulin-sensitizing, and mitochondrial-supportive properties [73,74,75]. *Tinospora cordifolia* has been reported to exert antioxidant, immunomodulatory, mitochondrial, and glucose-regulatory effects [76,77,78,79,80]. The pathways shown represent mechanisms reported in experimental or clinical studies and should not be interpreted as demonstrating direct causality between individual natural products and diabetic complications.

In this section, we critically evaluate selected natural products and functional foods for which experimental and/or clinical studies have reported potential effects on metabolic dysfunction associated with diabetes mellitus. These examples are summarized in Figure 3.

### 5.1. Curcumin and Turmeric

Curcumin, the principal bioactive polyphenolic constituent of *Curcuma longa* (turmeric), has attracted considerable scientific attention because of its broad spectrum of biological and pharmacological activities [18,19,81]. Turmeric has a long history of use in traditional medicine, while advances in molecular and cellular biology have demonstrated that curcumin can modulate multiple signaling pathways involved in metabolic regulation [18,19,82]. Curcumin exhibits antioxidant and anti-inflammatory activities and has been reported to influence pathways associated with insulin sensitivity, glucose and lipid metabolism, and cellular stress responses [18,19,82]. These pleiotropic actions provide a mechanistic rationale for investigating curcumin as a potential adjunctive intervention in diabetes; however, its clinical application is complicated by limited oral bioavailability and variability in formulations and clinical evidence [81].

#### 5.1.1. Chemical Properties and Biological Availability

Curcumin belongs to the diarylheptanoid family of polyphenolic compounds and contains two aromatic phenolic rings connected by a conjugated seven-carbon linker, a structural arrangement that contributes to its redox and antioxidant properties [18,19,83]. Curcumin can directly scavenge reactive oxygen and nitrogen species and can also interact with diverse molecular targets, including transcription factors, enzymes, kinases, and other signaling proteins involved in cellular stress responses [18,19,81].

Despite its therapeutic promise, clinical translation of curcumin remains challenging because of poor aqueous solubility, limited intestinal absorption, rapid metabolism, and extensive first-pass elimination [81,84]. To overcome these limitations, several strategies-including lipid-based formulations, nanoparticles, phospholipid complexes, micelles, and other bioavailability-enhancing formulations have been developed [85,86]. Although these approaches substantially improve systemic exposure, further pharmacokinetic optimization is still required before curcumin can be widely adopted in clinical practice.

#### 5.1.2. Antioxidant and Redox-Modulating Actions

One of the most extensively investigated properties of curcumin is its ability to modulate cellular redox homeostasis. In addition to directly scavenging reactive oxygen and nitrogen species, curcumin can enhance endogenous antioxidant defenses through activation of the nuclear factor erythroid 2-related factor 2 (Nrf2) signaling pathway, promoting the expression of antioxidant and cytoprotective enzymes [18,19,47]. Curcumin treatment has also been associated with increased activity or expression of antioxidant enzymes, including superoxide dismutase (SOD), catalase, and glutathione peroxidase (GPx), together with preservation of cellular glutathione homeostasis [47]. These coordinated effects can strengthen cellular resistance to oxidative injury under conditions of metabolic and oxidative stress [47,82].

Curcumin also influences mitochondrial redox homeostasis and function. Experimental studies suggest that curcumin can modulate mitochondrial respiration, reduce excessive mitochondrial ROS generation, and preserve mitochondrial membrane integrity under conditions of oxidative and metabolic stress [47]. In experimental models of diabetes, these antioxidant effects have been associated with reduced pancreatic β-cell oxidative injury and apoptosis, preservation of islet function, and improved insulin secretion [87]. Collectively, these findings suggest that modulation of mitochondrial dysfunction and oxidative stress represents an important component of the potential antidiabetic actions of curcumin.

#### 5.1.3. Modulation of Inflammatory Signaling Pathways

Persistent activation of inflammatory signaling contributes significantly to insulin resistance, and curcumin has consistently been shown to suppress several of these pathways [18,19,82]. One of the most consistently reported signaling effects of curcumin is modulation of the NF-κB pathway. Experimental studies suggest that curcumin may attenuate NF-κB activation and nuclear translocation, with associated reductions in the expression of inflammatory mediators such as TNF-α and IL-6 [18,19,82].

Beyond NF-κB, curcumin attenuates signaling through stress-activated kinases such as c-Jun N-terminal kinase (JNK), limiting inhibitory phosphorylation of insulin receptor substrate (IRS) proteins and preserving downstream insulin signaling [19,82,88]. Experimental studies have also reported reduced activation of inflammasome components and decreased macrophage infiltration within adipose tissue following curcumin administration [89,90]. Collectively, these anti-inflammatory effects contribute to improved metabolic homeostasis in obesity-associated diabetes.

#### 5.1.4. Effects on Insulin Sensitivity and Glucose Metabolism

Preclinical studies have demonstrated that curcumin can improve glucose homeostasis in several experimental models of obesity and diabetes, with reported reductions in fasting glucose and insulin resistance and improvements in glucose tolerance [18,19,48,91]. These metabolic effects have been associated with modulation of insulin-signaling pathways, including PI3K-Akt signaling and glucose transporter expression and trafficking, although the magnitude and direction of these effects can vary according to tissue, experimental model, dose, and treatment conditions [48,92].

Curcumin has also been reported to influence hepatic glucose and lipid metabolism by modulating pathways involved in gluconeogenesis, glycogen metabolism, and insulin sensitivity. In addition, regulation of metabolic targets such as peroxisome proliferator-activated receptor-γ (PPARγ) may contribute to its effects on glucose and lipid homeostasis [48,93]. Collectively, these findings suggest that curcumin can influence multiple pathways involved in glucose homeostasis and insulin sensitivity rather than acting solely through a direct glucose-lowering mechanism.

#### 5.1.5. Protection of Pancreatic β-Cell Function

Loss of functional pancreatic β-cell mass is a critical feature of diabetes progression. Experimental evidence suggests that curcumin has been associated with β-cell-protective effects in experimental models through multiple mechanisms, including attenuating oxidative stress, suppressing inflammatory signaling, and modulating endoplasmic reticulum (ER) stress [18,19,72]. In experimental models, curcumin has been associated with reduced β-cell apoptosis, improved islet-cell survival, and preservation of insulin secretory function [87,94].

Curcumin has also been reported to protect pancreatic islets against oxidative and glycation-associated injury and to preserve islet structure and function in experimental models [87,94]. By limiting oxidative, inflammatory, and ER-stress-associated β-cell injury, curcumin may help preserve β-cell viability and secretory capacity. However, these β-cell-protective effects have been demonstrated predominantly in cellular and animal models, and their clinical relevance requires further investigation [49].

#### 5.1.6. Effect on Lipid Metabolism and Obesity-Associated Diabetes

Dyslipidemia frequently accompanies type 2 diabetes and contributes to insulin resistance and cardiovascular complications. Curcumin has consistently demonstrated favorable effects on lipid metabolism in both experimental models and clinical investigations [50,95]. Supplementation reduces circulating triglycerides, total cholesterol, and low-density lipoprotein cholesterol while increasing high-density lipoprotein cholesterol [95].

These metabolic improvements are partly attributable to reduced free fatty acid accumulation and suppression of lipid-induced inflammatory signaling [24]. Curcumin additionally decreases macrophage infiltration into adipose tissue and improves adipokine secretion by increasing adiponectin while lowering leptin expression [95,96,97]. Together, these effects contribute to improved systemic insulin sensitivity and reduced metabolic inflammation.

#### 5.1.7. Evidence from Clinical Studies

Clinical studies evaluating curcumin supplementation in individuals with type 2 diabetes have generally reported favorable outcomes. Randomized controlled trials have shown improvements in fasting glucose, glycated hemoglobin (HbA1c), insulin resistance indices, oxidative stress biomarkers, inflammatory mediators, and lipid profiles following curcumin administration [51,52,95,98]. These findings support observations obtained from experimental models and strengthen the biological rationale for clinical application.

Nevertheless, interpretation of existing clinical evidence requires caution. Considerable variability exists among studies with respect to formulation, dosage, treatment duration, participant characteristics, and study design [51,95]. Many investigations also include relatively small sample sizes, limiting statistical power and assessment of long-term efficacy. Although current findings are encouraging, larger multicenter trials using standardized formulations remain necessary before curcumin can be routinely incorporated into evidence-based diabetes management.

#### 5.1.8. Limitations and Translational Considerations

Despite extensive preclinical evidence supporting the biological activity of curcumin, several challenges continue to limit its clinical translation. Poor oral bioavailability, low aqueous solubility, rapid metabolism and elimination, uncertainty regarding optimal dosing, and substantial variation among curcumin formulations can influence systemic exposure and contribute to heterogeneity across clinical studies [81,85]. Furthermore, differences in study populations, treatment duration, background therapies, and outcome measures complicate direct comparison among clinical trials [51]. Addressing these limitations will require standardized and pharmacokinetically characterized formulations, together with rigorous pharmacokinetic, pharmacodynamic, and longer-term clinical investigations.

Rather than replacing established antidiabetic medications, curcumin is more appropriately considered a potential adjunctive intervention because of its ability to modulate multiple pathways associated with oxidative stress, inflammation, mitochondrial dysfunction, and metabolic regulation [18,19,49]. However, the promising mechanistic and clinical findings should be interpreted in the context of variability in formulation, bioavailability, and overall certainty of clinical evidence. Future research integrating molecular biomarkers, optimized drug-delivery technologies, standardized formulations, and adequately powered randomized controlled trials will be important for defining whether curcumin can provide clinically meaningful benefits as part of comprehensive diabetes management strategies [51,85].

### 5.2. Honey as a Functional Natural Product in Diabetes Mellitus

Honey has been consumed for centuries as both a nutrient and a traditional medicinal product. Although it is composed predominantly of carbohydrates, particularly fructose and glucose, honey also contains a complex mixture of bioactive constituents, including phenolic acids, flavonoids, amino acids, organic acids, vitamins, enzymes, and trace minerals [99,100,101]. The composition and concentration of these constituents vary substantially according to botanical and geographical origin, bee species, processing, and storage conditions [100].

Experimental and clinical research suggests that some types of honey may exert biological effects beyond their nutritional value, including antioxidant, anti-inflammatory, and metabolic activities [99,100,102]. These properties have stimulated interest in honey as a functional food in metabolic-disease research, including diabetes mellitus; however, its high carbohydrate content, compositional variability, and heterogeneous clinical evidence require careful consideration when evaluating its potential role in diabetes management [99,102].

#### 5.2.1. Composition and Glycemic Characteristics

The carbohydrate fraction of honey consists predominantly of fructose and glucose, with smaller amounts of sucrose, oligosaccharides, and other minor sugars [99,100]. However, the relative abundance of these carbohydrates varies according to botanical and geographical origin and contributes, together with other compositional factors, to differences in the glycemic properties of individual honeys [100]. Honey therefore exhibits a wide range of glycemic index (GI) values rather than a uniformly low GI [103,104]. Some honey varieties produce a lower postprandial glycemic response than glucose or sucrose, but this effect varies substantially according to honey type, carbohydrate composition, dose, and study population [53,99,103].

Beyond its carbohydrate profile, honey contains a broad spectrum of phytochemicals with antioxidant activity, particularly phenolic acids and flavonoids [100]. These polyphenolic constituents contribute substantially to the antioxidant capacity of honey and may influence redox-sensitive and inflammatory pathways [54]. Collectively, these compositional characteristics support consideration of honey as a biologically active food; however, its metabolic effects should be interpreted in the context of its substantial sugar content and the considerable compositional variability among different honey types [100].

#### 5.2.2. Antioxidant and Anti-Inflammatory Properties

One of the principal mechanisms underlying the beneficial effects of honey involves attenuation of oxidative stress. Experimental studies consistently demonstrate that honey supplementation enhances endogenous antioxidant defenses by increasing the activity of enzymes such as superoxide dismutase, catalase, and glutathione peroxidase while reducing lipid peroxidation in diabetic tissues [55,99]. These protective effects have been documented in organs commonly affected by diabetic complications, including the pancreas, liver, and kidneys.

Honey may also modulate inflammatory responses associated with chronic metabolic disease. Several studies have reported reductions in circulating inflammatory mediators following honey supplementation, accompanied by improvements in antioxidant capacity [105]. Because oxidative stress and inflammation reinforce one another during diabetes progression, simultaneous regulation of both processes may contribute to improved insulin sensitivity and reduced tissue injury.

#### 5.2.3. Effects on Glycemic Control in Preclinical Models

Preclinical studies indicate that honey can influence glucose metabolism under diabetic conditions. In chemically induced models of diabetes, honey supplementation lowers fasting blood glucose concentrations, improves glucose tolerance, and attenuates metabolic disturbances associated with chronic hyperglycemia [56,106,107]. These improvements are frequently accompanied by reductions in circulating triglycerides and low-density lipoprotein cholesterol together with increases in high-density lipoprotein cholesterol, suggesting broader effects on lipid metabolism [107].

Interestingly, when compared with equivalent caloric amounts of refined sugars such as fructose or sucrose, honey often produces superior metabolic outcomes [108]. Animals receiving honey generally exhibit lower oxidative stress, reduced lipid peroxidation, and better glycemic control despite similar carbohydrate intake. These observations indicate that the biological activity of honey is determined not only by its sugar content but also by the synergistic actions of its minor bioactive constituents [99,100].

#### 5.2.4. Synergistic Effects with Conventional Antidiabetic Drugs

An area of growing interest is the use of honey alongside established antidiabetic medications. Experimental evidence suggests that combining honey with drugs such as metformin or glibenclamide enhances improvements in glucose metabolism while simultaneously reducing oxidative stress more effectively than pharmacological treatment alone [107,109]. Enhanced antioxidant enzyme activity and improved lipid metabolism have also been observed in these combination studies [57,107].

These findings support the concept that honey may complement rather than replace conventional pharmacotherapy. By targeting oxidative stress and inflammatory pathways that are not fully addressed by glucose-lowering medications, honey could potentially contribute to a more comprehensive approach to diabetes management. Nevertheless, confirmation of these findings in larger clinical studies remains necessary before routine therapeutic recommendations can be made.

#### 5.2.5. Evidence from Human Clinical Studies

Human studies evaluating honey consumption in individuals with diabetes have produced encouraging, although not entirely consistent, findings. Controlled clinical trials generally report lower postprandial glycemic responses following honey intake compared with refined sugars [58]. Some investigations have additionally demonstrated modest improvements in fasting blood glucose, serum lipid profiles, inflammatory biomarkers, and body weight after regular consumption of natural honey [110,111].

However, interpretation of these studies requires caution because clinical outcomes vary considerably according to honey source, botanical origin, processing methods, dosage, intervention duration, and characteristics of the study population [110]. Since honey remains a concentrated source of carbohydrates, excessive consumption may adversely affect glycemic control [111,112,113]. Consequently, any potential therapeutic use should be individualized and incorporated into an overall dietary strategy rather than viewed as unrestricted sugar substitution.

#### 5.2.6. Mechanistic Considerations and Controversies

The beneficial actions of honey likely result from the combined activity of multiple constituents rather than a single dominant compound. Proposed mechanisms include delayed intestinal glucose absorption, modulation of the gut microbiota through naturally occurring oligosaccharides, enhancement of insulin secretion, and attenuation of oxidative stress-induced insulin resistance [99,100]. Polyphenols present in honey may further regulate redox-sensitive transcription factors and inflammatory signaling pathways involved in metabolic homeostasis [54,99,100].

Despite these promising mechanisms, the use of honey in diabetes remains debated because of its carbohydrate content. The challenge lies in balancing its nutritional composition with its biologically active components. Current evidence suggests that moderate, carefully controlled intake may provide metabolic benefits, whereas excessive consumption is unlikely to be advantageous. Accordingly, honey should be regarded as a functional food that requires appropriate dietary context rather than as a universal therapeutic agent [105,110].

#### 5.2.7. Translational Implications

The complexity of honey illustrates both the opportunities and challenges associated with functional foods in metabolic disease. Its biological effects appear to arise from interactions among sugars, antioxidants, and numerous minor phytochemicals, making mechanistic interpretation more challenging than for isolated compounds [99,100]. Nevertheless, this complexity may also explain its ability to influence multiple metabolic pathways simultaneously.

Future investigations should emphasize standardized honey preparations, precise characterization of bioactive constituents, and adequately powered clinical trials incorporating biomarkers of oxidative stress, inflammation, mitochondrial function, and insulin signaling [110,114]. Such studies will be essential for determining whether honey can be incorporated into evidence-based nutritional strategies for the prevention and management of diabetes mellitus [105,110].

#### 5.2.8. Complex Food Matrices and Functional Foods

Natural bioactive constituents are frequently consumed within complex food matrices rather than as isolated compounds, and interactions among food components may influence their stability, bioaccessibility, absorption, metabolism, and biological activity. Functional foods such as fermented dairy products, yogurt-based preparations, plant-based fermented foods, and functional beverages may contain combinations of polyphenols, proteins, lipids, dietary fiber, vitamins, minerals, microbial metabolites, and, in some cases, viable microorganisms [115]. Fermentation may further modify phytochemical composition, generate additional bioactive metabolites, and influence interactions with the gut microbiota. These combined effects may produce metabolic responses that differ from those observed with isolated compounds. However, the presence of multiple bioactive components should not automatically be interpreted as evidence of synergistic activity, because interactions may be synergistic, additive, neutral, or antagonistic and therefore require direct experimental validation [116,117].

Fermented dairy products provide a useful illustration of these matrix-dependent effects. Randomized controlled trials of probiotic yogurt in individuals with T2DM have reported improvements in selected glycemic, lipid, and antioxidant parameters, although findings have not been uniform across studies. For example, probiotic yogurt containing Lactobacillus and Bifidobacterium species has been associated with improvements in HbA1c, lipid parameters, and antioxidant status in some clinical trials, whereas meta-analytic evidence has shown inconsistent effects on fasting glucose, insulin, and HOMA-IR. These findings highlight that the metabolic effects of complex functional foods may depend on microbial strain, food matrix, formulation, dose, intervention duration, and characteristics of the study population [116,118].

### 5.3. Antidiabetic Effects of Selected Spices: Modulation of Metabolic and Redox Pathways

Dietary spices have been used for centuries not only to enhance flavor but also for their medicinal properties in traditional healthcare systems [119,120]. Increasing experimental and clinical evidence suggests that many commonly consumed spices contain bioactive phytochemicals capable of influencing metabolic pathways involved in diabetes mellitus [119,121]. Unlike conventional pharmacological agents that generally act on specific molecular targets, spices contain multiple constituents that collectively influence glucose metabolism, lipid homeostasis, oxidative stress, and inflammatory signaling [119]. Although the magnitude and clinical relevance of these effects vary among individual spices, regular dietary consumption may provide supportive metabolic benefits as part of a broader lifestyle-based approach to diabetes management [121].

#### 5.3.1. Garlic (*Allium sativum*)

Garlic (*Allium sativum*) is among the most extensively studied dietary interventions for cardiometabolic disorders. Its biological activity is largely attributed to sulfur-containing compounds, including allicin, S-allyl cysteine, and various diallyl sulfides, which possess potent antioxidant and anti-inflammatory properties [59,60]. These compounds have been shown to influence multiple metabolic processes associated with diabetes, including insulin secretion, lipid metabolism, and oxidative stress [59,61].

Experimental studies consistently demonstrate that garlic supplementation lowers fasting blood glucose concentrations while improving serum lipid profiles in diabetic animal models [59]. These metabolic improvements are accompanied by reduced lipid peroxidation and enhanced activity of endogenous antioxidant enzymes, indicating restoration of redox homeostasis. Clinical investigations have likewise reported modest reductions in fasting glucose, triglycerides, and total cholesterol following garlic supplementation, particularly among individuals with type 2 diabetes or coexisting dyslipidemia [61,62]. Collectively, these findings suggest that garlic may provide cardiovascular as well as metabolic benefits when incorporated into long-term dietary management.

#### 5.3.2. Fenugreek (*Trigonella foenum-graceum*)

Fenugreek (*Trigonella foenum-graecum*) is widely investigated for its potential role in diabetes management because of its diverse bioactive constituents, including soluble fiber, saponins, alkaloids, and the unusual amino acid derivative 4-hydroxyisoleucine [65,66,67,68,69,70,122]. These constituents may influence several aspects of glucose and lipid metabolism through complementary mechanisms, including modulation of carbohydrate absorption, insulin secretion, insulin sensitivity, and cellular antioxidant defenses [65,67,70,122].

Clinical studies in individuals with diabetes have reported improvements in glycemic control following fenugreek supplementation, including reductions in fasting glucose, postprandial glucose, and HbA1c [66,68,69,123]. Recent meta-analyses support these glycemic effects, although findings regarding insulin resistance and individual lipid parameters remain variable across studies [123,124]. Experimental investigations further suggest that fenugreek may delay intestinal carbohydrate absorption, enhance peripheral glucose utilization, stimulate glucose-dependent insulin secretion, and strengthen antioxidant defenses [65,67,70,122]. Collectively, these findings support the potential of fenugreek as a multitarget dietary intervention, while differences in preparation, dosage, treatment duration, and study design should be considered when interpreting its clinical efficacy.

#### 5.3.3. Cumin (*Cuminum cyminum*)

Cumin (*Cuminum cyminum*) has attracted increasing attention because of its antioxidant and hypolipidemic properties. Experimental studies using diabetic animal models demonstrate reductions in blood glucose, glycated hemoglobin, plasma cholesterol, and triglycerides following cumin supplementation [125]. These metabolic improvements are accompanied by decreased oxidative stress, suggesting that enhancement of cellular redox balance contributes to its biological activity [126].

The therapeutic potential of cumin appears to arise primarily from its phenolic compounds and essential oils, which improve lipid metabolism while protecting cells against oxidative injury [125,126]. Although clinical evidence remains relatively limited, the consistency of findings from preclinical studies supports further evaluation of cumin as a complementary nutritional strategy for diabetes management.

#### 5.3.4. Coriander (*Coriandrum sativum*)

Coriander (*Coriandrum sativum*), commonly consumed worldwide as both a culinary herb and spice, has demonstrated beneficial effects on lipid metabolism and oxidative stress in experimental models of metabolic disease. Supplementation has been associated with reduced lipid peroxidation, improved antioxidant enzyme activity, and favorable changes in serum lipid metabolism [127,128,129]. Experimental studies in diabetic animals have further demonstrated reductions in blood glucose, total cholesterol, triglycerides, and low-density lipoprotein cholesterol, together with improvements in antioxidant defenses [129,130].

Unlike several other medicinal plants, coriander appears to exert its metabolic effects primarily through improvement of lipid handling and antioxidant capacity rather than through direct enhancement of insulin secretion [127,128,129]. Recent clinical evidence also suggests potential benefits in individuals with type 2 diabetes, with coriander seed supplementation producing improvements in glycemic indices, insulin resistance, lipid profiles, and oxidative stress biomarkers [131]. Nevertheless, additional well-designed clinical trials are needed before definitive therapeutic recommendations can be established.

#### 5.3.5. Ginger (*Zingiber officinale*)

The medicinal properties of ginger (*Zingiber officinale*) are largely attributed to bioactive compounds such as gingerols and shogaols, which possess potent antioxidant, anti-inflammatory, and lipid-regulating activities [132,133]. Experimental studies have shown that ginger supplementation reduces oxidative stress, improves lipid profiles, and attenuates vascular injury in models of metabolic disease [134].

Although the direct glucose-lowering effects of ginger remain variable across clinical studies, its ability to suppress oxidative stress and chronic inflammation may indirectly enhance insulin sensitivity and reduce diabetes-associated complications [134,135]. Consequently, ginger may be particularly valuable as part of a broader dietary strategy aimed at improving long-term metabolic health rather than achieving immediate glycemic control [134,136].

#### 5.3.6. Cinnamon (*Cinnamomum verum*)

Among culinary spices, cinnamon (*Cinnamomum verum*) has received considerable attention for its potential effects on glucose metabolism. Clinical studies have reported reductions in fasting blood glucose, triglycerides, and low-density lipoprotein cholesterol following cinnamon supplementation in individuals with type 2 diabetes [137,138]. Experimental investigations suggest that these metabolic benefits result from enhanced insulin receptor signaling, increased glucose uptake, and inhibition of carbohydrate-digesting enzymes [139].

In addition to its effects on glucose metabolism, cinnamon possesses antioxidant properties that may reduce oxidative stress and inflammatory signaling. However, the clinical literature remains heterogeneous because of differences in cinnamon species, preparation methods, dosage, treatment duration, and participant characteristics [137,140]. Standardization of study protocols will therefore be essential to determine the true therapeutic value of cinnamon in diabetes management.

#### 5.3.7. Integrated Perspective on Spices as Metabolic Modulators

Although each spice contains distinct bioactive constituents, several common mechanistic themes emerge. Most exhibit antioxidant and anti-inflammatory activities, improve lipid metabolism, and enhance insulin sensitivity to varying degrees [121,141]. Rather than acting through a single molecular target, these natural products influence interconnected metabolic pathways that collectively contribute to improved glucose homeostasis and reduced oxidative injury.

From a translational standpoint, dietary spices should be regarded as supportive nutritional interventions rather than substitutes for established pharmacological therapy. Their widespread culinary use and diverse bioactive composition make them attractive candidates for inclusion within broader dietary strategies for metabolic health; however, differences in preparation, dosage, bioavailability, and potential interactions with medications must be considered [141,142]. Future clinical studies should prioritize standardized preparations, clearly defined dosing regimens, and mechanistic biomarkers to better define the contribution of individual spices to evidence-based diabetes care [121].

### 5.4. Traditional Formulations with Antidiabetic Potential: Shilajit and Guduchi

Traditional systems of medicine have long utilized complex herbal and mineral preparations for the management of metabolic disorders. Unlike purified phytochemicals, these preparations contain diverse bioactive constituents that may influence multiple biological pathways simultaneously. Increasing experimental evidence indicates that several traditional remedies possess antioxidant, anti-inflammatory, and metabolic regulatory properties relevant to diabetes mellitus [76,120]. Among these, shilajit and Guduchi (*Tinospora cordifolia*) have attracted scientific interest because of their reported effects on glucose metabolism, oxidative stress, and other metabolic processes. However, much of the available evidence remains preclinical, and robust clinical evidence supporting their efficacy in diabetes is limited. Further mechanistic and well-designed clinical studies are therefore required to establish their efficacy, safety, optimal dosing, and potential role in diabetes management.

#### 5.4.1. Shilajit

Shilajit is a naturally occurring phyto-mineral substance found in mountainous regions, particularly the Himalayas. Chemically, it is a heterogeneous mixture containing humic substances, including fulvic acids, together with minerals, amino acids, phenolic compounds, and other low-molecular-weight organic constituents [73]. Dibenzo-α-pyrones and fulvic acid have also been proposed as important bioactive components of shilajit [74]. However, its precise chemical composition varies substantially according to geographical origin and processing, complicating identification of the constituents responsible for specific biological activities.

Preclinical studies have suggested potential antioxidant and metabolic effects of shilajit, including modulation of oxidative stress and lipid metabolism. However, compared with other natural products discussed in this review, evidence directly demonstrating antidiabetic effects remains limited, and many proposed metabolic benefits are derived from animal studies or broader pharmacological investigations rather than robust clinical trials [74]. Consequently, claims regarding improvements in glucose homeostasis, pancreatic β-cell protection, and antioxidant defenses should presently be interpreted as preliminary rather than established therapeutic effects.

Although antioxidant and cytoprotective mechanisms have been proposed, the evidence supporting direct preservation of pancreatic β-cell function by shilajit remains predominantly preclinical. Similarly, evidence supporting additive antidiabetic effects when shilajit is combined with conventional glucose-lowering medications is insufficient to establish an adjunctive therapeutic role. Well-designed mechanistic and clinical studies are therefore needed to determine whether these proposed effects translate into meaningful improvements in glycemic control in individuals with diabetes.

An additional translational challenge is the substantial variability in shilajit composition associated with geographical origin, purification, and processing. Recent analytical studies have also highlighted the presence of numerous metals, including potentially toxic elements, emphasizing the importance of product purification, compositional characterization, and rigorous quality control [73,75]. Greater characterization of its active constituents together with rigorous quality-control measures will therefore be essential before widespread clinical application can be considered.

#### 5.4.2. Guduchi

*Tinospora cordifolia*, commonly known as guduchi or giloy, is an important medicinal plant in Ayurvedic medicine and has traditionally been used for the management of various disorders, including diabetes and inflammatory conditions [77,120,143]. Phytochemical analyses have identified numerous bioactive constituents, including alkaloids, diterpenoid lactones, glycosides, polysaccharides, steroids, and phenolic compounds, several of which have been associated with antioxidant, anti-inflammatory, and immunomodulatory activities [78,143].

Preclinical studies have reported antihyperglycemic and metabolic effects of guduchi extracts in experimental models of diabetes. These effects include modulation of glucose and lipid metabolism together with enhancement of endogenous antioxidant defenses [77,79]. Experimental evidence further suggests that the antioxidant and cytoprotective properties of guduchi may reduce oxidative injury and apoptosis under diabetogenic conditions, providing a potential mechanism for preservation of pancreatic cellular function [79]. However, these findings remain predominantly preclinical and should not be interpreted as established β-cell protective effects in humans.

Several mechanisms have been proposed to explain these biological effects. Antioxidant activity may attenuate oxidative stress, while anti-inflammatory and immunomodulatory actions may influence cellular pathways involved in metabolic dysfunction [77,143]. Polysaccharides and other phytochemical constituents of guduchi have also demonstrated antioxidant, immunomodulatory, and antidiabetic activities in experimental investigations [78]. Collectively, these findings suggest that guduchi may influence multiple biological processes relevant to diabetes, although the relative contribution of individual constituents and pathways remains incompletely characterized.

Despite promising preclinical findings, robust clinical evidence supporting the efficacy of guduchi for diabetes remains limited. Furthermore, safety requires careful consideration because clinically apparent liver injury has been reported in association with guduchi consumption. Consequently, future investigations should employ botanically authenticated and chemically standardized preparations, rigorous safety monitoring, and well-designed randomized clinical trials to establish efficacy, optimal dosage, and long-term safety before *T. cordifolia* can be considered for evidence-based diabetes management.

#### 5.4.3. Comparative and Translational Considerations

Both shilajit and guduchi illustrate the potential of traditional medicinal preparations to influence multiple biological processes relevant to complex metabolic diseases. Rather than acting through a single molecular target, their diverse constituents have been associated with modulation of oxidative stress, inflammatory responses, glucose and lipid metabolism, and cellular protective mechanisms [74,77]. Such multitarget activity is potentially relevant to diabetes, where disease progression involves interconnected disturbances in metabolic, redox, and inflammatory pathways. However, much of the evidence supporting these effects remains preclinical, and the strength of evidence differs considerably between individual preparations and proposed mechanisms.

Nevertheless, translation of these traditional remedies into evidence-based clinical practice requires careful evaluation. Standardization and authentication of source materials, identification and characterization of active constituents, rigorous quality control, and adequately powered randomized clinical trials remain essential prerequisites for determining their therapeutic relevance [73,143]. Safety evaluation is equally important, particularly given concerns regarding compositional variability and potential contaminants in shilajit and reports of liver injury associated with guduchi consumption [75,80]. Integrating traditional medicinal knowledge with contemporary molecular, pharmacological, and clinical research may ultimately help determine whether standardized preparations can be safely incorporated as complementary interventions in diabetes management.

The experimental and clinical evidence, major bioactive constituents, proposed mechanisms, and metabolic effects of the natural products discussed in this review are summarized in Table 1 and Table 2.

## 6. Limitations, Knowledge Gaps, and Translational Challenges

Although numerous experimental and clinical studies have investigated the therapeutic potential of natural products in diabetes mellitus, several important challenges continue to limit their translation into routine clinical practice. Available evidence suggests potential effects on glucose metabolism, oxidative stress, inflammation, and lipid homeostasis; however, the strength and consistency of these findings vary substantially among individual natural products [141,142]. Considerable heterogeneity in study design, product composition and standardization, dosage, intervention duration, participant characteristics, and methodological quality further complicate interpretation and comparison of the existing literature. Addressing these limitations will be essential for determining whether specific natural products can be safely and effectively incorporated as evidence-based complementary interventions in diabetes management. Inconsistencies among studies may partly reflect differences in botanical species, cultivar, geographical origin, cultivation and harvesting conditions, extraction procedures, processing, storage, purity, phytochemical composition, formulation, dose, bioavailability, intervention duration, and participant characteristics. Consequently, findings obtained with one preparation should not automatically be extrapolated to all products marketed under the same botanical or common name. Detailed chemical characterization and standardized reporting are therefore essential for meaningful comparison across studies.

### 6.1. Variability and Lack of Standardization

One of the greatest obstacles in natural product research is the considerable variability among preparations. The chemical composition of medicinal plants, spices, honey, and traditional formulations is influenced by numerous factors, including botanical origin, geographical location, cultivation practices, harvesting conditions, storage, processing, and extraction methods [100,146]. Consequently, preparations that share the same common name may differ substantially in their phytochemical composition, concentrations of active constituents, and biological activity.

This inconsistency makes comparison across experimental and clinical studies difficult and may contribute to heterogeneous or conflicting outcomes. Reliable evaluation of therapeutic efficacy therefore requires rigorous authentication of source materials, standardized extraction and processing procedures, comprehensive phytochemical characterization, and implementation of robust quality-control measures [114,146]. Establishing well-defined and reproducible quality standards would substantially improve consistency across studies and facilitate meaningful comparison of findings from independent investigations.

### 6.2. Bioavailability and Pharmacokinetic Constraints

Limited bioavailability remains a major barrier to the clinical application of many natural compounds. Curcumin represents one of the best-known examples, as poor aqueous solubility, limited gastrointestinal absorption, rapid metabolism, and systemic elimination significantly reduce its bioavailability following oral administration [147,148]. Several advanced formulations—including nanoparticles, phospholipid complexes, liposomes, and lipid-based delivery systems—have been developed to improve curcumin solubility, stability, absorption, and systemic exposure. Although these approaches have improved pharmacokinetic properties, formulation-dependent differences remain an important consideration when interpreting therapeutic efficacy across clinical studies.

Bioavailability also presents challenges for the bioactive constituents of functional foods such as honey and dietary spices. The bioaccessibility, absorption, metabolism, and biological activity of dietary phytochemicals can be influenced by food processing, gastrointestinal digestion, and interactions with the gut microbiota [149,150]. Gut microbial metabolism is particularly important for many dietary polyphenols because it generates metabolites that may differ substantially in absorption and biological activity from their parent compounds [151]. These factors, together with inter-individual differences in gut microbial composition and metabolism, may contribute to variability in biological responses and complicate interpretation of clinical outcomes [152]. Administered dose does not necessarily reflect systemic exposure to biologically active constituents [153]. Poor solubility, limited intestinal absorption, first-pass metabolism, rapid elimination, food-matrix effects, formulation differences, and microbial metabolism can substantially influence bioavailability [150]. These factors may explain some of the apparent inconsistencies in efficacy across studies and complicate direct dose-to-dose comparisons among different preparations [154].

### 6.3. Statistical Significance Versus Clinical Relevance

Statistical significance should not automatically be interpreted as clinically meaningful benefit. Changes in fasting glucose, HbA1c, lipid parameters, inflammatory markers, or oxidative-stress biomarkers should be interpreted in relation to the magnitude of effect, baseline metabolic status, intervention duration, comparator treatment, background pharmacotherapy, and confidence intervals. Small statistically significant changes in surrogate biomarkers may indicate biological activity without establishing meaningful therapeutic benefit. Longer and adequately powered clinical studies are needed to determine whether such changes translate into sustained improvements in glycemic control or reductions in clinically relevant diabetes outcomes.

### 6.4. Limitations of Preclinical Models

A substantial proportion of mechanistic knowledge regarding natural products has been generated using in vitro systems and experimental animal models. While these approaches have been invaluable for identifying molecular targets and biological pathways, they do not fully replicate the complexity and heterogeneity of human diabetes [155,156]. Differences in metabolism, immune regulation, genetic background, pancreatic physiology, gut microbiota, and disease progression can limit the direct translation of findings from laboratory models to clinical practice [155,157].

Another important consideration is that some preclinical studies employ doses or exposure conditions that may not correspond directly to those achievable through normal dietary intake or routine supplementation in humans [158]. Differences in treatment duration and experimental protocols further complicate translation, with many animal studies being relatively short-term and varying substantially in dose, administration route, and outcome measures [159]. Future preclinical investigations should therefore incorporate physiologically relevant dosing strategies, appropriate dose translation, standardized experimental designs, and longer-term intervention protocols that more closely reflect human disease progression and therapeutic use.

### 6.5. Heterogeneity and Design Limitations in Clinical Studies

Clinical investigations evaluating natural products for diabetes management remain highly heterogeneous. Many published studies involve relatively small cohorts, short intervention periods, and substantial variability in participant characteristics, product formulation, dosage, treatment duration, background therapy, and clinical endpoints [141]. Such methodological differences make direct comparison across studies difficult and reduce confidence in pooled estimates and the overall evidence base.

Furthermore, most clinical trials emphasize conventional metabolic outcomes such as fasting blood glucose, HbA1c, insulin resistance indices, and lipid parameters, while mechanistic biomarkers are assessed less consistently. Incorporation of validated biomarkers reflecting oxidative stress, inflammatory signaling, insulin sensitivity, and other relevant molecular pathways could improve mechanistic interpretation of clinical responses. Future research should therefore prioritize adequately powered, longer-duration, multicenter randomized controlled trials using standardized and chemically characterized interventions, clearly defined outcomes, and rigorous safety monitoring [141].

### 6.6. Safety, Interactions, and Regulatory Considerations

Although natural products are frequently perceived as inherently safe, their clinical use warrants careful evaluation. Herbal formulations and dietary supplements may interact with conventional antidiabetic medications, potentially altering therapeutic effects or increasing the risk of adverse events [142]. Product contamination or adulteration with heavy metals, pesticides, microbial or fungal contaminants, toxins, or undeclared pharmacologically active substances also represents an important safety concern [160,161]. These considerations emphasize the importance of appropriate quality control, product authentication, and safety monitoring.

Regulatory classification further complicates their clinical application. Dietary and herbal supplements are regulated differently from pharmaceutical products, and regulatory frameworks, manufacturing requirements, quality-control practices, and labeling standards vary across jurisdictions [162]. These differences can contribute to variability in product quality and complicated assessment of safety and efficacy. Strengthening evidence-based regulatory frameworks, analytical quality-control methods, and post-market safety surveillance will therefore be important for ensuring product quality, patient safety, and appropriate clinical use.

Commercial products may differ substantially from preparations evaluated in published studies with respect to identity, purity, potency, formulation, and bioavailability. Adulteration, contamination, substitution of botanical material, inaccurate labeling, and undeclared active ingredients represent additional concerns, particularly in poorly regulated or online markets. Findings obtained with a research-grade or standardized preparation should therefore not automatically be extrapolated to commercially available supplements carrying the same ingredient name.

Regulatory frameworks for natural products and dietary supplements differ across jurisdictions and may influence product quality, labeling, and consumer safety. In the United States, dietary supplements are regulated under the Dietary Supplement Health and Education Act of 1994 (DSHEA), with manufacturing requirements addressed under Current Good Manufacturing Practice regulations for dietary supplements (21 CFR Part 111). In the European Union, food supplements are regulated principally under Directive 2002/46/EC, while nutrition and health claims are governed by Regulation (EC) No 1924/2006. Despite these frameworks, commercially available products may differ substantially in composition, purity, dose, formulation, and bioavailability. Therefore, evidence obtained using standardized research preparations should not automatically be extrapolated to all commercially available products marketed under the same ingredient name.

### 6.7. Future Directions/Artificial Intelligence and Data-Driven Approach

Future research should move beyond demonstrating efficacy toward understanding the molecular mechanisms that determine therapeutic response. Integration of metabolomics, transcriptomics, proteomics, systems biology, and precision nutrition approaches may provide a more comprehensive understanding of how natural products influence complex metabolic networks associated with diabetes. These technologies also offer opportunities to identify biomarkers that predict responsiveness to specific interventions and facilitate personalized therapeutic strategies.

**Figure 4:**
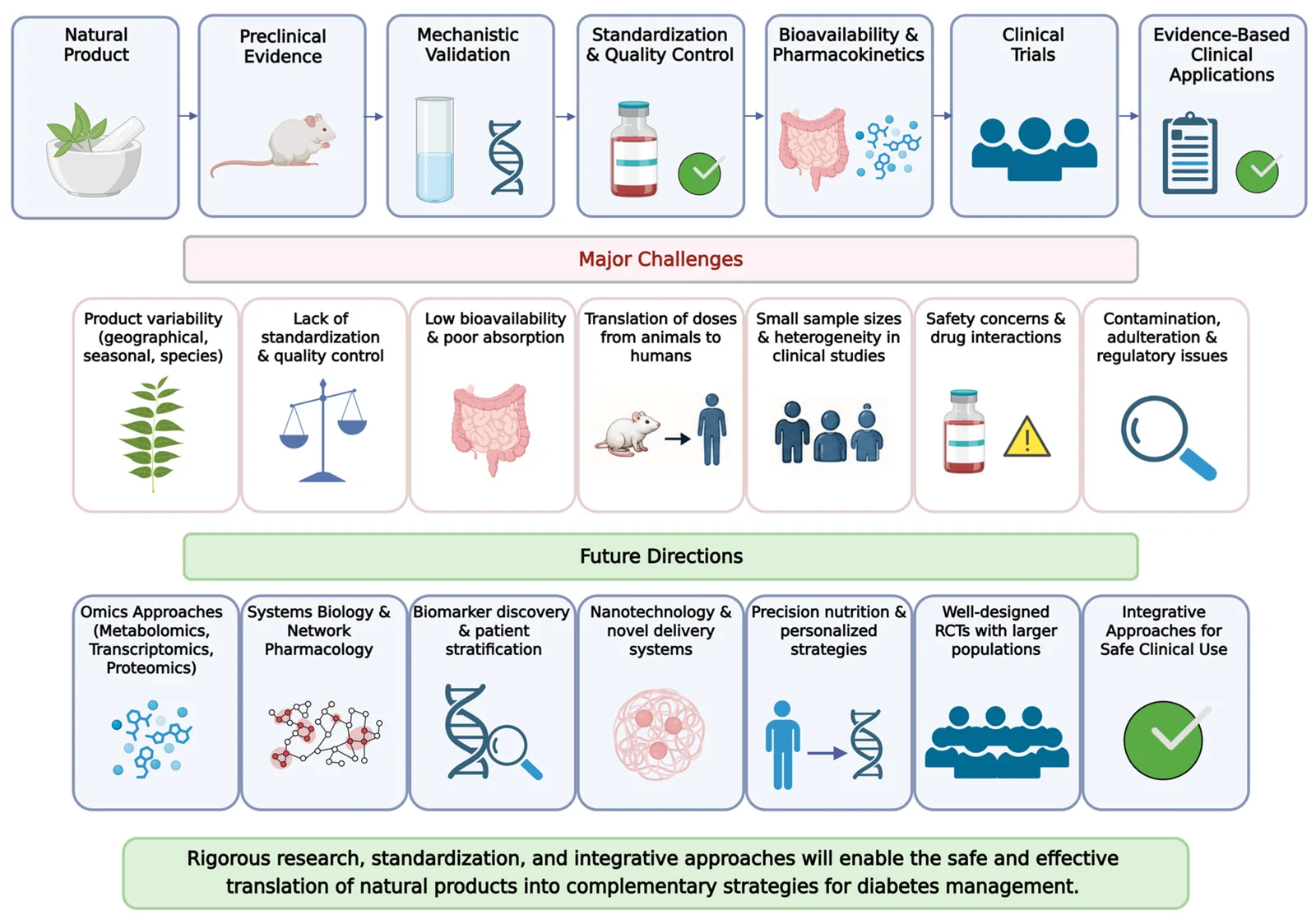
Summary of the major translational challenges and future research priorities for advancing natural products toward evidence-based diabetes management.

Equally important will be the development of standardized formulations supported by rigorous pharmacokinetic studies and well-designed multicenter clinical trials. Combining traditional medicinal knowledge with modern molecular biology, pharmacology, and clinical research has the potential to accelerate the translation of promising natural compounds into scientifically validated adjunctive therapies for diabetes mellitus. The major translational challenges and future research priorities for advancing natural products toward evidence-based diabetes management are summarized in Figure 4.

Artificial intelligence and machine-learning approaches may facilitate natural-product research through phytochemical screening, prediction of biological activity, integration of multi-omics datasets, toxicity assessment, and prioritization of candidate compounds for experimental investigation [163]. However, such approaches should be regarded as hypothesis-generating tools and cannot replace experimental validation, pharmacokinetic assessment, safety testing, or clinical trials [164].

## 7. Conclusions and Future Perspectives

Diabetes mellitus is a multifactorial metabolic disease characterized not only by chronic hyperglycemia but also by profound disturbances in lipid metabolism, mitochondrial function, oxidative stress, and persistent low-grade inflammation. These interconnected pathological processes contribute to progressive β-cell dysfunction, insulin resistance, and the development of microvascular and macrovascular complications. Consequently, therapeutic strategies directed solely at lowering blood glucose are unlikely to fully address the complex biological mechanisms responsible for disease progression.

The evidence summarized in this review indicates that naturally derived bioactive compounds possess considerable potential as complementary interventions for diabetes management. Compounds and functional foods such as curcumin, honey, garlic, fenugreek, cumin, coriander, ginger, and cinnamon influence multiple pathways involved in metabolic regulation, including oxidative stress, inflammatory signaling, glucose and lipid metabolism, and insulin sensitivity. Traditional preparations such as shilajit and guduchi have also demonstrated potentially relevant biological activities, although their evidence base remains predominantly preclinical and requires further clinical validation. Collectively, the multitarget properties of these natural products provide a biologically plausible rationale for continued investigation alongside established therapeutic approaches.

Despite encouraging findings from experimental studies and an increasing number of clinical investigations, important challenges remain before these natural products can be routinely incorporated into evidence-based clinical practice. Variability in chemical composition, limited bioavailability, inconsistent study designs, and the scarcity of large, well-controlled clinical trials continue to restrict definitive conclusions regarding efficacy and optimal therapeutic use. Addressing these limitations through standardized formulations, rigorous pharmacokinetic evaluation, and carefully designed multicenter studies will be essential for translating promising laboratory findings into meaningful clinical applications.

Future advances in systems biology, metabolomics, precision nutrition, and molecular pharmacology are expected to provide deeper insight into how natural compounds interact with complex metabolic networks. Integrating these emerging technologies with traditional medicinal knowledge may facilitate the identification of novel therapeutic targets, improve patient stratification, and support the development of personalized adjunctive treatment strategies. Such an approach has the potential to maximize therapeutic efficacy while minimizing variability in clinical outcomes.

In conclusion, selected natural products and functional foods may influence metabolic, oxidative, inflammatory, mitochondrial, and endocrine pathways relevant to diabetes mellitus; however, the strength and clinical relevance of the evidence vary considerably among products and formulations. Biological plausibility, favorable preclinical findings, or statistically significant changes in surrogate biomarkers should not be considered equivalent to established clinical efficacy. Natural products should therefore be regarded as potential adjunctive approaches rather than replacements for evidence-based diabetes prevention and treatment. Future research should emphasize standardized formulations, rigorous chemical characterization, pharmacokinetic assessment, adequately powered clinical trials, clinically meaningful outcomes, and long-term safety.

## Figures and Tables

**Table 1:** Summary of Experimental/Pre-Clinical Evidence, Proposed Mechanisms, and Metabolic Effects of Selected Natural Products in Diabetes Mellitus.

Natural Product	Experimental/Preclinical Evidence	Cell/Tissue Model	Animal Species/Models	Principal Mechanisms	Key Outcomes	Current Evidence and Key Limitations	Ref.
**Curcumin (** ***Curcuma longa*** **)**	STZ- and alloxan-induced diabetic models; diet-induced obesity models; clinical studies in T2DM	3T3-L1 adipocytes Adipose tissue INS-1 pancreatic β-cell line Pancreatic β-cells Pancreatic islets/β-cell injury Metabolic tissues	Male mice Hamsters	Antioxidant and anti-inflammatory activity; modulation of NF-κB/JNK signaling; activation of Nrf2-related antioxidant defenses; modulation of insulin signaling and glucose/lipid metabolism	Improvements reported in glycemic and lipid parameters; reduced oxidative stress; potential cellular protective effects	Preclinical + clinical evidence; poor native bioavailability and substantial formulation/trial heterogeneity	[51,95]
**Honey**	Experimental diabetic models; controlled human studies including T2DM	Pancreatic cell lines	Rat	Antioxidant and anti-inflammatory activity; polyphenol-mediated effects; potential modulation of glucose/lipid metabolism and gut-related mechanisms	Variable effects on fasting glucose and lipid parameters; antioxidant/metabolic effects reported	Preclinical + clinical evidence, but heterogeneous; effects depend on floral source, processing, dose, and population; excessive intake remains a concern because honey is carbohydrate-rich	[99,111]
**Garlic (** ***Allium sativum*** **)**	Experimental diabetic models; clinical studies in T2DM and dyslipidemia	3T3 L1 adipocytes	Wistar Rats	Organosulfur-mediated antioxidant and anti-inflammatory effects; modulation of glucose and lipid metabolism	Reduced fasting glucose and improvements in lipid parameters reported; reduced oxidative stress in experimental studies	Preclinical + clinical evidence; variation in garlic preparation, dose, and intervention duration	[61,62]
**Fenugreek (** ***Trigonella foenum-graecum*** **)**	Diabetic animal models; clinical studies in T1DM and T2DM	3T3 L1 adipocytes	Rats	Delayed carbohydrate absorption; soluble-fiber effects; modulation of insulin secretion/sensitivity; antioxidant activity	Improvements in fasting/postprandial glucose and lipid parameters; reduced insulin resistance reported	Preclinical + clinical evidence; substantial variation in seed/extract preparations and dosage	[123,124]
**Cumin (** ***Cuminum cyminum*** **)**	Experimental diabetic models; limited clinical studies	-	Rats	Antioxidant activity; modulation of lipid metabolism; phenolic and essential-oil-associated effects	Reduced glucose, HbA1c, and lipid parameters reported in some studies	Preclinical + limited clinical evidence; relatively small and heterogeneous clinical evidence base	[125,126]
**Coriander (** ***Coriandrum sativum*** **)**	Primarily experimental metabolic/diabetic models	-	-	Reduced lipid peroxidation; enhancement of antioxidant enzymes; modulation of lipid metabolism	Reduced triglycerides/cholesterol and increased HDL reported in experimental studies	Predominantly preclinical evidence; insufficient high-quality human trials	[129,131]
**Ginger (** ***Zingiber officinale*** **)**	Experimental metabolic models; human supplementation studies	Pancreatic B cells	Rats	Gingerol/shogaol-associated antioxidant and anti-inflammatory effects; modulation of lipid and glucose metabolism	Improvements in oxidative stress, lipid and glycemic parameters reported	Preclinical + clinical evidence; heterogeneity in formulation, dosage and clinical outcomes	[134,136]
**Cinnamon (** ***Cinnamomum verum*** **)**	Experimental models; clinical studies in T2DM	3T3L1 adipocytes	Rats	Modulation of insulin signaling and glucose uptake; inhibition of carbohydrate-digesting enzymes; antioxidant activity	Improvements in fasting glucose and some lipid parameters reported	Preclinical + clinical evidence, but heterogeneous; cinnamon species, preparation, dose and duration differ substantially among studies	[137,140]
**Shilajit**	Primarily experimental/preclinical investigations	-	-	Proposed antioxidant and cytoprotective actions; modulation of oxidative stress and lipid metabolism	Potential improvements in metabolic and oxidative-stress parameters reported preclinically	Predominantly preclinical evidence; insufficient robust diabetes clinical trials; major compositional, standardization and quality-control concerns	[73,75]
**Guduchi (*Tinospora cordifolia*)**	Predominantly experimental diabetic models; limited human evidence	3T3L1 adipocytes	Rats	Antioxidant, anti-inflammatory, and immunomodulatory activity; potential modulation of glucose/lipid metabolism and cellular protection	Antihyperglycemic and metabolic effects reported primarily in experimental studies	Predominantly preclinical/limited clinical evidence; extract standardization is needed, and liver-injury safety concerns require consideration	[77,79]

Abbreviations: STZ, streptozotocin; T1DM, type 1 diabetes mellitus; T2DM, type 2 diabetes mellitus; NF-κB, nuclear factor kappa-light-chain-enhancer of activated B cells; Nrf2, nuclear factor erythroid 2-related factor 2; HbA1c, glycated hemoglobin; HDL, high-density lipoprotein.

**Table 2:** Clinical Evidence for Selected Natural Products and Functional Foods in Diabetes Mellitus.

Product	Study/Design	Population	Formulation/Dose	Duration	Primary Outcomes	Safety/Limitations	Reference
Curcumin	randomized, double-blind, placebo-controlled add-on trial	70 adults with T2DM	Nano-curcumin micelles, 80 mg/day	3 months	HbA1c, fasting glucose, lipid profile, BMI	Small single-center trial; nano-formulation limits extrapolation to conventional curcumin; short duration.	[144]
Honey	randomized controlled crossover trial	53 adults with T2DM enrolled; 42 completed	Natural honey, 50 g/day added to a weight-maintenance diet	8 weeks per phase; 4-week washout	HbA1c, fasting glucose, insulin, anthropometric measures	High carbohydrate load is clinically relevant; crossover study was relatively short; results caution against assuming glycemic benefit from honey.	[111,113]
Garlic	systematic review and meta-analysis of RCTs	8 randomized clinical studies in adults with T2DM	Garlic preparations varied across trials	Varied across included studies	Fasting glucose, HbA1c, total cholesterol, LDL-C, HDL-C, triglycerides	Very high heterogeneity for glycemic and lipid outcomes; products, doses, and durations differed across trials.	[61]
Fenugreek	double-blind placebo-controlled clinical trial	25 newly diagnosed adults with mild-to-moderate T2DM	Hydroalcoholic fenugreek seed extract, 1 g/day	2 months	Fasting and post-glucose glucose, insulin, HbA1c, lipids, HOMA-IR	Small sample; short duration; extract-specific findings may not generalize to whole seed or other preparations.	[66]
Coriander	randomized, double-blind, placebo-controlled trial	40 adults with T2DM, age 30–60 years	Coriander seed powder, 1000 mg/day	6 weeks	Fasting glucose, insulin, HOMA-IR, lipid profile, MDA, total antioxidant capacity	Small sample and short duration; single formulation; longer and larger trials are needed.	[131]
Ginger	randomized, double-blind, placebo-controlled trial	41 adults with T2DM	Ginger powder, 2 g/day	12 weeks	Fasting glucose, HbA1c, ApoB, ApoA-I, malondialdehyde	Small sample; short intervention; powdered ginger formulation; clinical effect size and long-term relevance require confirmation.	[145]
Cinnamon	systematic review and dose–response meta-analysis of RCTs	24 RCTs in adults with T2DM	Cinnamon preparations and doses varied across trials	Varied across included studies	Fasting glucose, HbA1c, HOMA-IR, insulin	Substantial heterogeneity in species, dose, preparation, duration, background therapy, and study quality limits direct clinical extrapolation.	[138]

## Data Availability

No data was generated for writing of this review. All information is contained within the manuscript.

## Abbreviations

The following abbreviations are used in this manuscript:

AGEs	Advanced glycation end products
AP-1	Activator protein-1
ATP	Adenosine triphosphate
CAT	Catalase
CRP	C-reactive protein
ER	Endoplasmic reticulum
GLUT4	Glucose transporter type 4
GPx	Glutathione peroxidase
GR	Glutathione reductase
HbA1c	Glycated hemoglobin
HDL	High-density lipoprotein
IKK	Inhibitor of κB kinase
IRS	Insulin receptor substrate
JNK	c-Jun N-terminal kinase
LDL	Low-density lipoprotein
NADPH	Nicotinamide adenine dinucleotide phosphate
NF-κB	Nuclear factor kappa B
Nrf2	Nuclear factor erythroid 2-related factor 2
PI3K	Phosphoinositide 3-kinase
PPARγ	Peroxisome proliferator-activated receptor gamma
RAGE	Receptor for advanced glycation end products
RNS	Reactive nitrogen species
ROS	Reactive oxygen species
SGLT2	Sodium-glucose cotransporter 2
SOD	Superoxide dismutase
STZ	Streptozotocin
T1DM	Type 1 diabetes mellitus
T2DM	Type 2 diabetes mellitus
UPR	Unfolded protein response

## References

[1] Genitsaridi I., Salpea P., Salim A., Sajjadi S.F., Tomic D., James S., Thirunavukkarasu S., Issaka A., Chen L., Basit A. (2026). 11th edition of the IDF Diabetes Atlas: Global, regional, and national diabetes prevalence estimates for 2024 and projections for 2050. Lancet Diabetes Endocrinol..

[2] Federation I.D. (2025). IDF Global Clinical Practice Recommendations for the Management of Type 2 Diabetes.

[3] American Diabetes Association Professional Practice Committee (2025). 2. Diagnosis and Classification of Diabetes: Standards of Care in Diabetes-2025. Diabetes Care.

[4] Mauvais F.-X., van Endert P.M. (2025). Type 1 Diabetes: A Guide to Autoimmune Mechanisms for Clinicians. Diabetes Obes. Metab..

[5] Feng Z., Tan Z., Lu D. (2025). Mitochondrial bioenergetics dysfunction in T2DM: Linking oxidative stress to insulin resistance. Front. Endocrinol..

[6] Islam K., Islam R., Nguyen I., Malik H., Pirzadah H., Shrestha B., Lentz I.B., Shekoohi S., Kaye A.D. (2025). Diabetes Mellitus and Associated Vascular Disease: Pathogenesis, Complications, and Evolving Treatments. Adv. Ther..

[7] Accili D., Deng Z., Liu Q. (2025). Insulin resistance in type 2 diabetes mellitus. Nat. Rev. Endocrinol..

[8] Yau B., Ghislain J., Kebede M.A., Hughes J., Poitout V. (2025). The role of the beta cell in type 2 diabetes: New findings from the last 5 years. Diabetologia.

[9] Evans J.L., Goldfine I.D., Maddux B.A., Grodsky G.M. (2002). Oxidative stress and stress-activated signaling pathways: A unifying hypothesis of type 2 diabetes. Endocr. Rev..

[10] Chen X., Xie N., Feng L., Huang Y., Wu Y., Zhu H., Tang J., Zhang Y. (2025). Oxidative stress in diabetes mellitus and its complications: From pathophysiology to therapeutic strategies. Chin. Med. J..

[11] Sajithlal G.B., Chithra P., Chandrakasan G. (1998). Effect of curcumin on the advanced glycation and cross-linking of collagen in diabetic rats. Biochem. Pharmacol..

[12] Yang D.-R., Wang M.-Y., Zhang C.-L., Wang Y. (2024). Endothelial dysfunction in vascular complications of diabetes: A comprehensive review of mechanisms and implications. Front. Endocrinol..

[13] Shoelson S.E., Lee J., Yuan M. (2003). Inflammation and the IKK beta/I kappa B/NF-kappa B axis in obesity- and diet-induced insulin resistance. Int. J. Obes. Relat. Metab. Disord..

[14] Qin L., Fan B., Zhou Y., Zheng J., Diao R., Wang F., Liu J. (2025). Targeted gut microbiome therapy: Applications and prospects of probiotics, fecal microbiota transplantation and natural products in the management of type 2 diabetes. Pharmacol. Res..

[15] Nikola L., Iva L. (2024). Gut microbiota as a modulator of type 1 diabetes: A molecular perspective. Life Sci..

[16] American Diabetes Association Professional Practice Committee (2025). 9. Pharmacologic Approaches to Glycemic Treatment: Standards of Care in Diabetes-2025. Diabetes Care.

[17] Sharma H., Chandola H.M. (2011). Prameha in Ayurveda: Correlation with obesity, metabolic syndrome, and diabetes mellitus. Part 2--management of Prameha. J. Altern. Complement. Med..

[18] Gupta S.C., Sung B., Kim J.H., Prasad S., Li S., Aggarwal B.B. (2013). Multitargeting by turmeric, the golden spice: From kitchen to clinic. Mol. Nutr. Food Res..

[19] Aggarwal B.B., Harikumar K.B. (2009). Potential therapeutic effects of curcumin, the anti-inflammatory agent, against neurodegenerative, cardiovascular, pulmonary, metabolic, autoimmune and neoplastic diseases. Int. J. Biochem. Cell Biol..

[20] Altobelli E., Angeletti P.M., Marziliano C., Mastrodomenico M., Giuliani A.R., Petrocelli R. (2021). Potential Therapeutic Effects of Curcumin on Glycemic and Lipid Profile in Uncomplicated Type 2 Diabetes-A Meta-Analysis of Randomized Controlled Trial. Nutrients.

[21] Bobiş O., Dezmirean D.S., Moise A.R. (2018). Honey and Diabetes: The Importance of Natural Simple Sugars in Diet for Preventing and Treating Different Type of Diabetes. Oxidative Med. Cell. Longev..

[22] Rohit S., Natália M., Ashun C., Neha G., Vineet S., Kamil K., Eugenie N., Hardeep Singh T., Anupam B., Anand C. (2020). Adjunct use of honey in diabetes mellitus: A consensus or conundrum?. Trends Food Sci. Technol..

[23] Sultan Ayoub M., Mohammad Javed A., Kamran S., Habib Ullah C., Waseem H., Saleh A. (2017). Honey and diabetes mellitus: Obstacles and challenges—Road to be repaired. Saudi J. Biol. Sci..

[24] Weinberg Sibony R., Segev O., Dor S., Raz I. (2024). Overview of oxidative stress and inflammation in diabetes. J. Diabetes.

[25] Lenzen S. (2008). The mechanisms of alloxan- and streptozotocin-induced diabetes. Diabetologia.

[26] Radenković M., Stojanović M., Prostran M. (2016). Experimental diabetes induced by alloxan and streptozotocin: The current state of the art. J. Pharmacol. Toxicol. Methods.

[27] Campbell J.E., Drucker D.J. (2013). Pharmacology, physiology, and mechanisms of incretin hormone action. Cell Metab..

[28] Nauck M.A., Müller T.D. (2023). Incretin hormones and type 2 diabetes. Diabetologia.

[29] Soodini G.R. (2004). Adiponectin and leptin in relation to insulin sensitivity. Metab. Syndr. Relat. Disord..

[30] Cryer P.E. (2005). Mechanisms of hypoglycemia-associated autonomic failure and its component syndromes in diabetes. Diabetes.

[31] Elkanawati R.Y., Sumiwi S.A., Levita J. (2024). Impact of Lipids on Insulin Resistance: Insights from Human and Animal Studies. Drug Des. Devel Ther..

[32] Nguyen M.T.A., Hiroaki S., Svetlana F., Jennie L.B., Takeshi I., Juan I.S., Jonathan Z., Bassil I.D., Nai-Wen C., Jerrold M.O. (2005). JNK and Tumor Necrosis Factor-α Mediate Free Fatty Acid-induced Insulin Resistance in 3T3-L1 Adipocytes. J. Biol. Chem..

[33] Gaita L., Timar B., Timar R., Fras Z., Gaita D., Banach M. (2024). Lipid Disorders Management Strategies (2024) in Prediabetic and Diabetic Patients. Pharmaceuticals.

[34] Plötz T., Lenzen S. (2024). Mechanisms of lipotoxicity-induced dysfunction and death of human pancreatic beta cells under obesity and type 2 diabetes conditions. Obes. Rev..

[35] Montgomery M.K., Turner N. (2015). Mitochondrial dysfunction and insulin resistance: An update. Endocr. Connect..

[36] Darwish R., Alcibahy Y., Abu-Sharia G., Butler A.E. (2025). β-Cell Mitochondrial Dysfunction: Underlying Mechanisms and Potential Therapeutic Strategies. Cells.

[37] Zaher A., Stephens S.B. (2025). Breaking the Feedback Loop of β-Cell Failure: Insight into the Pancreatic β-Cell’s ER-Mitochondria Redox Balance. Cells.

[38] Singh H., Singh R., Singh A., Singh H., Singh G., Kaur S., Singh B. (2024). Role of oxidative stress in diabetes-induced complications and their management with antioxidants. Arch. Physiol. Biochem..

[39] Holendová B., Benáková Š., Křivonosková M., Plecitá-Hlavatá L. (2024). Redox Status as a Key Driver of Healthy Pancreatic Beta-Cells. Physiol. Res..

[40] Victoria R., Ivan N., Ulf S. (2003). Interleukin-6 (IL-6) Induces Insulin Resistance in 3T3-L1 Adipocytes and Is, Like IL-8 and Tumor Necrosis Factor-α, Overexpressed in Human Fat Cells from Insulin-resistant Subjects. J. Biol. Chem..

[41] Ying W., Fu W., Lee Y.S., Olefsky J.M. (2020). The role of macrophages in obesity-associated islet inflammation and β-cell abnormalities. Nat. Rev. Endocrinol..

[42] Diane A., Allouch A., Mu U.M.R.B.A., Al-Siddiqi H.H. (2024). Endoplasmic reticulum stress in pancreatic β-cell dysfunctionality and diabetes mellitus: A promising target for generation of functional hPSC-derived β-cells in vitro. Front. Endocrinol..

[43] Ansari P., Khan J.T., Chowdhury S., Reberio A.D., Kumar S., Seidel V., Abdel-Wahab Y.H.A., Flatt P.R. (2024). Plant-Based Diets and Phytochemicals in the Management of Diabetes Mellitus and Prevention of Its Complications: A Review. Nutrients.

[44] Cheng J., Li J., Xiong R.G., Wu S.X., Xu X.Y., Tang G.Y., Huang S.Y., Zhou D.D., Li H.B., Feng Y. (2024). Effects and mechanisms of anti-diabetic dietary natural products: An updated review. Food Funct..

[45] Ramasamy R., Yan S.F., Schmidt A.M. (2011). Receptor for AGE (RAGE): Signaling mechanisms in the pathogenesis of diabetes and its complications. Ann. N. Y. Acad. Sci..

[46] Li H., Meng Y., He S., Tan X., Zhang Y., Zhang X., Wang L., Zheng W. (2022). Macrophages, Chronic Inflammation, and Insulin Resistance. Cells.

[47] Avendaño-Briseño K.A., Escutia-Martínez J., Hernández-Cruz E.Y., Pedraza-Chaverri J. (2025). Antioxidant Effect of Curcumin and Its Impact on Mitochondria: Evidence from Biological Models. J. Xenobiot..

[48] Bozkurt O., Kocaadam-Bozkurt B., Yildiran H. (2022). Effects of curcumin, a bioactive component of turmeric, on type 2 diabetes mellitus and its complications: An updated review. Food Funct..

[49] Campagna R., Cecati M., Vignini A. (2025). The Multifaceted Role of the Polyphenol Curcumin: A Focus on Type 2 Diabetes Mellitus. Curr. Diabetes Rev..

[50] Jang E.M., Choi M.S., Jung U.J., Kim M.J., Kim H.J., Jeon S.M., Shin S.K., Seong C.N., Lee M.K. (2008). Beneficial effects of curcumin on hyperlipidemia and insulin resistance in high-fat-fed hamsters. Metabolism.

[51] Bahari H., Malekahmadi M., Shahraki Jazinaki M., Asadi Z., Soltaninejad K., Golafrouz H. (2026). Effects of Curcumin/Turmeric Supplementation on Lipid Profile in Subjects With Prediabetes and Diabetes: A Systematic Review and Meta-Analysis. Lipids.

[52] Chuengsamarn S., Rattanamongkolgul S., Phonrat B., Tungtrongchitr R., Jirawatnotai S. (2014). Reduction of atherogenic risk in patients with type 2 diabetes by curcuminoid extract: A randomized controlled trial. J. Nutr. Biochem..

[53] Abdulrhman M., El-Hefnawy M., Hussein R., El-Goud A.A. (2011). The glycemic and peak incremental indices of honey, sucrose and glucose in patients with type 1 diabetes mellitus: Effects on C-peptide level-a pilot study. Acta Diabetol..

[54] Wilczyńska A., Żak N. (2024). Polyphenols as the Main Compounds Influencing the Antioxidant Effect of Honey-A Review. Int. J. Mol. Sci..

[55] El-Aarag B., Shehata S.B., El-Garawani I.M., El-Seedi H.R., Nofal A.E. (2024). Regulation of Oxidative Stress and Apoptosis in Streptozotocin-Induced Diabetic Rats by Egyptian Sidr Honey. Chem. Biodivers..

[56] Erejuwa O.O., Gurtu S., Sulaiman S.A., Ab Wahab M.S., Sirajudeen K.N., Salleh M.S. (2010). Hypoglycemic and antioxidant effects of honey supplementation in streptozotocin-induced diabetic rats. Int. J. Vitam. Nutr. Res..

[57] Erejuwa O.O., Sulaiman S.A., Wahab M.S., Salam S.K., Salleh M.S., Gurtu S. (2011). Comparison of antioxidant effects of honey, glibenclamide, metformin, and their combinations in the kidneys of streptozotocin-induced diabetic rats. Int. J. Mol. Sci..

[58] Al-Waili N.S. (2004). Natural honey lowers plasma glucose, C-reactive protein, homocysteine, and blood lipids in healthy, diabetic, and hyperlipidemic subjects: Comparison with dextrose and sucrose. J. Med. Food.

[59] Padiya R., Banerjee S.K. (2013). Garlic as an anti-diabetic agent: Recent progress and patent reviews. Recent. Pat. Food Nutr. Agric..

[60] Ribeiro M., Alvarenga L., Cardozo L., Chermut T.R., Sequeira J., de Souza Gouveia Moreira L., Teixeira K.T.R., Shiels P.G., Stenvinkel P., Mafra D. (2021). From the distinctive smell to therapeutic effects: Garlic for cardiovascular, hepatic, gut, diabetes and chronic kidney disease. Clin. Nutr..

[61] Ebrahimzadeh A., Safargar M., Rajabzadeh-Dehkordi M., Nematolahi F., Samadani M., Saeid L., Mohtashamian A., Izadi M.A., Ebrahimzadeh A., Safarpour A.R. (2026). The Therapeutic Effect of Garlic Supplements on the Metabolic Profile of Patients With Type 2 Diabetes: A Systematic Review and Meta-Analysis of Randomized Clinical Trials. Nutr. Metab. Insights.

[62] Zhao X., Cheng T., Xia H., Yang Y., Wang S. (2024). Effects of Garlic on Glucose Parameters and Lipid Profile: A Systematic Review and Meta-Analysis on Randomized Controlled Trials. Nutrients.

[63] Bordia A., Verma S.K., Srivastava K.C. (1998). Effect of garlic (*Allium sativum*) on blood lipids, blood sugar, fibrinogen and fibrinolytic activity in patients with coronary artery disease. Prostaglandins Leukot. Essent. Fat. Acids.

[64] Sobenin I.A., Nedosugova L.V., Filatova L.V., Balabolkin M.I., Gorchakova T.V., Orekhov A.N. (2008). Metabolic effects of time-released garlic powder tablets in type 2 diabetes mellitus: The results of double-blinded placebo-controlled study. Acta Diabetol..

[65] Devi B.A., Kamalakkannan N., Prince P.S. (2003). Supplementation of fenugreek leaves to diabetic rats. Effect on carbohydrate metabolic enzymes in diabetic liver and kidney. Phytother. Res..

[66] Gupta A., Gupta R., Lal B. (2001). Effect of Trigonella foenum-graecum (fenugreek) seeds on glycaemic control and insulin resistance in type 2 diabetes mellitus: A double blind placebo controlled study. J. Assoc. Physicians India.

[67] Hamden K., Masmoudi H., Carreau S., Elfeki A. (2010). Immunomodulatory, beta-cell, and neuroprotective actions of fenugreek oil from alloxan-induced diabetes. Immunopharmacol. Immunotoxicol..

[68] Kassaian N., Azadbakht L., Forghani B., Amini M. (2009). Effect of fenugreek seeds on blood glucose and lipid profiles in type 2 diabetic patients. Int. J. Vitam. Nutr. Res..

[69] Sharma R.D., Raghuram T.C., Rao N.S. (1990). Effect of fenugreek seeds on blood glucose and serum lipids in type I diabetes. Eur. J. Clin. Nutr..

[70] Zafar M.I., Gao F. (2016). 4-Hydroxyisoleucine: A Potential New Treatment for Type 2 Diabetes Mellitus. BioDrugs.

[71] Shabil M., Bushi G., Bodige P.K., Maradi P.S., Patra B.P., Padhi B.K., Khubchandani J. (2023). Effect of Fenugreek on Hyperglycemia: A Systematic Review and Meta-Analysis. Medicina.

[72] Cao Z., Wu Z., Duan T., Tang C., Huang D., Hu X. (2022). Curcumin ameliorates HO-induced injury through SIRT1-PERK-CHOP pathway in pancreatic beta cells. Acta Biochim. Biophys. Sin..

[73] Kamgar E., Kaykhaii M., Zembrzuska J. (2025). A Comprehensive Review on Shilajit: What We Know about Its Chemical Composition. Crit. Rev. Anal. Chem..

[74] Stohs S.J. (2014). Safety and efficacy of shilajit (mumie, moomiyo). Phytother. Res..

[75] Hussain A., Saeed A. (2024). Hazardous or Advantageous: Uncovering the Roles of Heavy Metals and Humic Substances in Shilajit (Phyto-mineral) with Emphasis on Heavy Metals Toxicity and Their Detoxification Mechanisms. Biol. Trace Elem. Res..

[76] Panchabhai T.S., Kulkarni U.P., Rege N.N. (2008). Validation of therapeutic claims of Tinospora cordifolia: A review. Phytother. Res..

[77] Sharma P., Dwivedee B.P., Bisht D., Dash A.K., Kumar D. (2019). The chemical constituents and diverse pharmacological importance of *Tinospora cordifolia*. Heliyon.

[78] Kumar M., Hasan M., Sharma A., Suhag R., Maheshwari C., Radha, Chandran D., Sharma K., Dhumal S., Senapathy M. (2023). *Tinospora cordifolia* (Willd.) Hook.f. & Thomson polysaccharides: A review on extraction, characterization, and bioactivities. Int. J. Biol. Macromol..

[79] Kalekar S.A., Munshi R.P., Thatte U.M. (2013). Do plants mediate their anti-diabetic effects through anti-oxidant and anti-apoptotic actions? an in vitro assay of 3 Indian medicinal plants. BMC Complement. Altern. Med..

[80] Kulkarni A.V., Hanchanale P., Prakash V., Kalal C., Sharma M., Kumar K., Bishnu S., Kulkarni A.V., Anand L., Patwa A.K. (2022). Tinospora Cordifolia (Giloy)-Induced Liver Injury During the COVID-19 Pandemic-Multicenter Nationwide Study From India. Hepatol. Commun..

[81] El-Saadony M.T., Saad A.M., Mohammed D.M., Alkafaas S.S., Ghosh S., Negm S.H., Salem H.M., Fahmy M.A., Mosa W.F.A., Ibrahim E.H. (2025). Curcumin, an active component of turmeric: Biological activities, nutritional aspects, immunological, bioavailability, and human health benefits—A comprehensive review. Front. Immunol..

[82] Zamanian M.Y., Alsaab H.O., Golmohammadi M., Yumashev A., Jabba A.M., Abid M.K., Joshi A., Alawadi A.H., Jafer N.S., Kianifar F. (2024). NF-κB pathway as a molecular target for curcumin in diabetes mellitus treatment: Focusing on oxidative stress and inflammation. Cell Biochem. Funct..

[83] Sun D.J., Zhu L.J., Zhao Y.Q., Zhen Y.Q., Zhang L., Lin C.C., Chen L.X. (2020). Diarylheptanoid: A privileged structure in drug discovery. Fitoterapia.

[84] Anand P., Kunnumakkara A.B., Newman R.A., Aggarwal B.B. (2007). Bioavailability of curcumin: Problems and promises. Mol. Pharm..

[85] Darmonkow A., Rowe Z.E.M., Harding S.V. (2025). Advancements in curcuminoid formulations: An update on bioavailability enhancement strategies curcuminoid bioavailability and formulations. Open Life Sci..

[86] Tabanelli R., Brogi S., Calderone V. (2021). Improving Curcumin Bioavailability: Current Strategies and Future Perspectives. Pharmaceutics.

[87] Duan J., Yang M., Liu Y., Xiao S., Zhang X. (2022). Curcumin protects islet beta cells from streptozotocin-induced type 2 diabetes mellitus injury via its antioxidative effects. Endokrynol. Pol..

[88] Priyanka A., Shyni G.L., Anupama N., Raj P.S., Anusree S.S., Raghu K.G. (2017). Development of insulin resistance through sprouting of inflammatory markers during hypoxia in 3T3-L1 adipocytes and amelioration with curcumin. Eur. J. Pharmacol..

[89] Islam T., Koboziev I., Albracht-Schulte K., Mistretta B., Scoggin S., Yosofvand M., Moussa H., Zabet-Moghaddam M., Ramalingam L., Gunaratne P.H. (2021). Curcumin Reduces Adipose Tissue Inflammation and Alters Gut Microbiota in Diet-Induced Obese Male Mice. Mol. Nutr. Food Res..

[90] Pandey S.N., Babu M.A., Goyal K., Menon S.V., Ray S., Kaur M., Sharma S., Rana M., Rekha A., Ali H. (2025). Targeting NLRP3 inflammasome with curcumin: Mechanisms and therapeutic promise in chronic inflammation. Inflammopharmacology.

[91] Hewlings S.J., Kalman D.S. (2017). Curcumin: A Review of Its Effects on Human Health. Foods.

[92] Song Z., Wang H., Zhu L., Han M., Gao Y., Du Y., Wen Y. (2015). Curcumin improves high glucose-induced INS-1 cell insulin resistance via activation of insulin signaling. Food Funct..

[93] Mohammadi E., Behnam B., Mohammadinejad R., Guest P.C., Simental-Mendía L.E., Sahebkar A. (2021). Antidiabetic Properties of Curcumin: Insights on New Mechanisms. Adv. Exp. Med. Biol..

[94] Meghana K., Sanjeev G., Ramesh B. (2007). Curcumin prevents streptozotocin-induced islet damage by scavenging free radicals: A prophylactic and protective role. Eur. J. Pharmacol..

[95] Dehzad M.J., Ghalandari H., Amini M.R., Askarpour M. (2023). Effects of curcumin/turmeric supplementation on lipid profile: A GRADE-assessed systematic review and dose-response meta-analysis of randomized controlled trials. Complement. Ther. Med..

[96] Musazadeh V., Abbasi S., Kavyani Z., Moridpour A.H., Safarzadeh D., Moradi Z., Bahadori F., Faghfouri A.H. (2024). The effect of curcumin supplementation on circulating adiponectin and leptin concentration in adults: A GRADE-assessed systematic review and meta-analysis of randomised controlled trials. Br. J. Nutr..

[97] Yamazaki Y., Kawano Y., Uebayasi M. (2008). Induction of adiponectin by natural and synthetic phenolamides in mouse and human preadipocytes and its enhancement by docosahexaenoic acid. Life Sci..

[98] Na L.X., Li Y., Pan H.Z., Zhou X.L., Sun D.J., Meng M., Li X.X., Sun C.H. (2013). Curcuminoids exert glucose-lowering effect in type 2 diabetes by decreasing serum free fatty acids: A double-blind, placebo-controlled trial. Mol. Nutr. Food Res..

[99] Erejuwa O.O., Sulaiman S.A., Wahab M.S. (2012). Honey--a novel antidiabetic agent. Int. J. Biol. Sci..

[100] Wang H., Li L., Lin X., Bai W., Xiao G., Liu G. (2023). Composition, functional properties and safety of honey: A review. J. Sci. Food Agric..

[101] Zammit Young G.W., Blundell R. (2023). A review on the phytochemical composition and health applications of honey. Heliyon.

[102] Bolat E., Canbolat A.A., Sarıtaş S., Lombardo M., Witkowska A.M., El-Seedi H., Bechelany M., Karav S. (2026). Honey as a Natural Flavorful Product: A Comprehensive Review of Its Potential Biological Activities and Recent Studies. Food Sci. Nutr..

[103] Atayoğlu A.T., Soylu M., Silici S., İnanç N. (2016). Glycemic index values of monofloral Turkish honeys and the effect oftheir consumption on glucose metabolism. Turk. J. Med. Sci..

[104] Bogdanov S., Jurendic T., Sieber R., Gallmann P. (2008). Honey for nutrition and health: A review. J. Am. Coll. Nutr..

[105] Norouzzadeh M., Barazandeh S., Hasan Rashedi M., Jamshidi S., Hatamifar F., Maghsoomi Z., Teymoori F., Malek M. (2025). Dosage exploration of the effects of honey and its derivatives on cardiometabolic outcomes: An overview of systematic reviews and GRADE-assessed updated meta-analysis. Nutr. Diabetes.

[106] Emeka N.N., Ghasi S.I., Sampson E., Erejuwa O.O. (2024). Effect of Honey on Postprandial Hyperglycemia in Alloxan-Induced Diabetic Rats. Arch. Razi Inst..

[107] Erejuwa O.O., Sulaiman S.A., Wahab M.S., Sirajudeen K.N., Salleh M.S., Gurtu S. (2011). Glibenclamide or metformin combined with honey improves glycemic control in streptozotocin-induced diabetic rats. Int. J. Biol. Sci..

[108] Busserolles J., Gueux E., Rock E., Mazur A., Rayssiguier Y. (2002). Substituting honey for refined carbohydrates protects rats from hypertriglyceridemic and prooxidative effects of fructose. J. Nutr..

[109] Erejuwa O.O., Sulaiman S.A., Wahab M.S., Salam S.K., Salleh M.S., Gurtu S. (2010). Antioxidant protective effect of glibenclamide and metformin in combination with honey in pancreas of streptozotocin-induced diabetic rats. Int. J. Mol. Sci..

[110] Ahmed A., Tul-Noor Z., Lee D., Bajwah S., Ahmed Z., Zafar S., Syeda M., Jamil F., Qureshi F., Zia F. (2023). Effect of honey on cardiometabolic risk factors: A systematic review and meta-analysis. Nutr. Rev..

[111] Sadeghi F., Salehi S., Kohanmoo A., Akhlaghi M. (2019). Effect of Natural Honey on Glycemic Control and Anthropometric Measures of Patients with Type 2 Diabetes: A Randomized Controlled Crossover Trial. Int. J. Prev. Med..

[112] Bahrami M., Ataie-Jafari A., Hosseini S., Foruzanfar M.H., Rahmani M., Pajouhi M. (2009). Effects of natural honey consumption in diabetic patients: An 8-week randomized clinical trial. Int. J. Food Sci. Nutr..

[113] Sadeghi F., Akhlaghi M., Salehi S. (2020). Adverse effects of honey on low-density lipoprotein cholesterol and adiponectin concentrations in patients with type 2 diabetes: A randomized controlled cross-over trial. J. Diabetes Metab. Disord..

[114] Majtan J. (2024). In vitro testing of honey quality and biological functionality: Underestimated elements in the clinical testing of honey. Front. Nutr..

[115] Mirjalili M., Salari Sharif A., Sangouni A.A., Emtiazi H., Mozaffari-Khosravi H. (2023). Effect of probiotic yogurt consumption on glycemic control and lipid profile in patients with type 2 diabetes mellitus: A randomized controlled trial. Clin. Nutr. ESPEN.

[116] Ejtahed H.S., Mohtadi-Nia J., Homayouni-Rad A., Niafar M., Asghari-Jafarabadi M., Mofid V. (2012). Probiotic yogurt improves antioxidant status in type 2 diabetic patients. Nutrition.

[117] Dehzad M.J., Raja A., Moghdani Z., Sohrabi Z., Fararooei M., Famouri M., Askarpour M., Babajafari S. (2025). Effects of Yogurt Enriched with Konjac Glucomannan and Inulin on Insulin Sensitivity, Glycemic Control, Lipid Profiles, Anthropometric Measures and Oxidative Stress in Type 2 Diabetes Mellitus: A Randomized Controlled Trial. Prev. Nutr. Food Sci..

[118] Barengolts E., Smith E.D., Reutrakul S., Tonucci L., Anothaisintawee T. (2019). The Effect of Probiotic Yogurt on Glycemic Control in Type 2 Diabetes or Obesity: A Meta-Analysis of Nine Randomized Controlled Trials. Nutrients.

[119] Mojica L., Luzardo-Ocampo I., Fonseca Hernandez D., Fu L., Zhang Q. (2026). Culinary Herbs and Spices: Uses Across the World, Nutritional and Health Benefits. Annu. Rev. Food Sci. Technol..

[120] Sharma H., Chandola H.M., Singh G., Basisht G. (2007). Utilization of Ayurveda in health care: An approach for prevention, health promotion, and treatment of disease. Part 1--Ayurveda, the science of life. J. Altern. Complement. Med..

[121] Garza M.C., Pérez-Calahorra S., Rodrigo-Carbó C., Sánchez-Calavera M.A., Jarauta E., Mateo-Gallego R., Gracia-Rubio I., Lamiquiz-Moneo I. (2024). Effect of Aromatic Herbs and Spices Present in the Mediterranean Diet on the Glycemic Profile in Type 2 Diabetes Subjects: A Systematic Review and Meta-Analysis. Nutrients.

[122] Annida B., Stanely Mainzen Prince P. (2005). Supplementation of fenugreek leaves reduces oxidative stress in streptozotocin-induced diabetic rats. J. Med. Food.

[123] Vajdi M., Noshadi N., Bonyadian A., Golpour-Hamedani S., Alipour B., Pourteymour Fard Tabrizi F., Abbasalizad-Farhangi M., Askari G. (2024). Therapeutic effect of fenugreek supplementation on type 2 diabetes mellitus: A systematic review and meta-analysis of clinical trials. Heliyon.

[124] Shakil S., Akhtar S.E., Ali A., Antony M., Antony I., Mansour E., Khawar Farooqui S., Akbar A., Alazazzi H., Alsufyani R. (2024). Enhancing glycaemic control and promoting cardiovascular health: The therapeutic potential of Trigonella foenumgraecum in diabetic patients—A systematic review and meta-analysis. Ann. Med. Surg..

[125] Dhandapani S., Subramanian V.R., Rajagopal S., Namasivayam N. (2002). Hypolipidemic effect of *Cuminum cyminum* L. on alloxan-induced diabetic rats. Pharmacol. Res..

[126] Singh N., Yadav S.S., Kumar S., Narashiman B. (2021). A review on traditional uses, phytochemistry, pharmacology, and clinical research of dietary spice *Cuminum cyminum* L.. Phytother. Res..

[127] Chithra V., Leelamma S. (1997). Hypolipidemic effect of coriander seeds (*Coriandrum sativum*): Mechanism of action. Plant Foods Hum. Nutr..

[128] Chithra V., Leelamma S. (1999). *Coriandrum sativum* changes the levels of lipid peroxides and activity of antioxidant enzymes in experimental animals. Indian J. Biochem. Biophys..

[129] Deepa B., Anuradha C.V. (2011). Antioxidant potential of *Coriandrum sativum* L. seed extract. Indian J. Exp. Biol..

[130] Sreelatha S., Inbavalli R. (2012). Antioxidant, antihyperglycemic, and antihyperlipidemic effects of *Coriandrum sativum* leaf and stem in alloxan-induced diabetic rats. J. Food Sci..

[131] Zamany S., Barzegar A., Aghapour B., Ferdosi F. (2025). Effects of Dietary Supplementation with Coriander Seed Powder on Serum Lipid Profile, Glycemic Indices, and Oxidative Stress Biomarkers in Patients with Type 2 Diabetes Mellitus: A Randomized, Double-Blind, Placebo-Controlled Trial. Prev. Nutr. Food Sci..

[132] Ayustaningwarno F., Anjani G., Ayu A.M., Fogliano V. (2024). A critical review of Ginger’s (*Zingiber officinale*) antioxidant, anti-inflammatory, and immunomodulatory activities. Front. Nutr..

[133] Maghraby Y.R., Labib R.M., Sobeh M., Farag M.A. (2023). Gingerols and shogaols: A multi-faceted review of their extraction, formulation, and analysis in drugs and biofluids to maximize their nutraceutical and pharmaceutical applications. Food Chem. X.

[134] Van B., Abdalla A.N., Algarni A.S., Khalid A., Zengin G., Aumeeruddy M.Z., Mahomoodally M.F. (2023). Zingiber officinale Roscoe (Ginger) and its Bioactive Compounds in Diabetes: A Systematic Review of Clinical Studies and Insight of Mechanism of Action. Curr. Med. Chem..

[135] Jalali M., Mahmoodi M., Moosavian S.P., Jalali R., Ferns G., Mosallanezhad A., Imanieh M.H., Mosallanezhad Z. (2020). The effects of ginger supplementation on markers of inflammatory and oxidative stress: A systematic review and meta-analysis of clinical trials. Phytother. Res..

[136] Schumacher J.C., Mueller V., Sousa C., Peres K.K., da Mata I.R., Menezes R.C.R., Dal Bosco S.M. (2024). The effect of oral supplementation of ginger on glycemic control of patients with type 2 diabetes mellitus—A systematic review and meta-analysis. Clin. Nutr. ESPEN.

[137] de Moura S.L., Gomes B.G.R., Guilarducci M.J., Coelho O.G.L., Guimarães N.S., Gomes J.M.G. (2025). Effects of cinnamon supplementation on metabolic biomarkers in individuals with type 2 diabetes: A systematic review and meta-analysis. Nutr. Rev..

[138] Moridpour A.H., Kavyani Z., Khosravi S., Farmani E., Daneshvar M., Musazadeh V., Faghfouri A.H. (2024). The effect of cinnamon supplementation on glycemic control in patients with type 2 diabetes mellitus: An updated systematic review and dose-response meta-analysis of randomized controlled trials. Phytother. Res..

[139] Silva M.L., Bernardo M.A., Singh J., de Mesquita M.F. (2022). Cinnamon as a Complementary Therapeutic Approach for Dysglycemia and Dyslipidemia Control in Type 2 Diabetes Mellitus and Its Molecular Mechanism of Action: A Review. Nutrients.

[140] Bakhach H., Nuffer M., Tall Bull S., Nuffer W. (2024). A Systematic Review Evaluating Cinnamon’s Effects on Glucose Utilizing a Ranking System to Assess Bias and Study Quality. J. Med. Food.

[141] Li E., Maunder A., Liu J., Pandey C., Cave A., O’Fee A., Ee C. (2025). The efficacy and safety of herbal medicines for glycaemic control and insulin resistance in individuals with type 2 diabetes: An umbrella review. BMC Complement. Med. Ther..

[142] Boulares E., Bragazzi N.L., Yin T.S.C., Choi S.J., Park J.H., Han D. (2026). Assessing the safety of herbal medicine use among type 2 diabetes mellitus patients: A systematic review and meta-analysis. Complement. Ther. Med..

[143] Singh D., Chaudhuri P.K. (2017). Chemistry and Pharmacology of *Tinospora cordifolia*. Nat. Prod. Commun..

[144] Rahimi H.R., Mohammadpour A.H., Dastani M., Jaafari M.R., Abnous K., Ghayour Mobarhan M., Kazemi Oskuee R. (2016). The effect of nano-curcumin on HbA1c, fasting blood glucose, and lipid profile in diabetic subjects: A randomized clinical trial. Avicenna J. Phytomed..

[145] Khandouzi N., Shidfar F., Rajab A., Rahideh T., Hosseini P., Mir Taheri M. (2015). The effects of ginger on fasting blood sugar, hemoglobin a1c, apolipoprotein B, apolipoprotein a-I and malondialdehyde in type 2 diabetic patients. Iran. J. Pharm. Res..

[146] Heinrich M., Jalil B., Abdel-Tawab M., Echeverria J., Kulić Ž., McGaw L.J., Pezzuto J.M., Potterat O., Wang J.-B. (2022). Best Practice in the chemical characterisation of extracts used in pharmacological and toxicological research—The ConPhyMP—Guidelines12. Front. Pharmacol..

[147] Dei Cas M., Ghidoni R. (2019). Dietary Curcumin: Correlation between Bioavailability and Health Potential. Nutrients.

[148] Khezri K., Saeedi M., Mohammadamini H., Zakaryaei A.S. (2021). A comprehensive review of the therapeutic potential of curcumin nanoformulations. Phytother. Res..

[149] Sejbuk M., Mirończuk-Chodakowska I., Karav S., Witkowska A.M. (2024). Dietary Polyphenols, Food Processing and Gut Microbiome: Recent Findings on Bioavailability, Bioactivity, and Gut Microbiome Interplay. Antioxidants.

[150] Williamson G. (2025). Bioavailability of Food Polyphenols: Current State of Knowledge. Annu. Rev. Food Sci. Technol..

[151] Favari C., Rinaldi de Alvarenga J.F., Sánchez-Martínez L., Tosi N., Mignogna C., Cremonini E., Manach C., Bresciani L., Del Rio D., Mena P. (2024). Factors driving the inter-individual variability in the metabolism and bioavailability of (poly)phenolic metabolites: A systematic review of human studies. Redox Biol..

[152] Espín J.C., González-Sarrías A., Tomás-Barberán F.A. (2017). The gut microbiota: A key factor in the therapeutic effects of (poly)phenols. Biochem. Pharmacol..

[153] Manach C., Williamson G., Morand C., Scalbert A., Rémésy C. (2005). Bioavailability and bioefficacy of polyphenols in humans. I. Review of 97 bioavailability studies. Am. J. Clin. Nutr..

[154] Visvanathan R., Williamson G. (2022). Review of factors affecting citrus polyphenol bioavailability and their importance in designing in vitro, animal, and intervention studies. Compr. Rev. Food Sci. Food Saf..

[155] Asrafuzzaman M., Cao Y., Afroz R., Kamato D., Gray S., Little P.J. (2017). Animal models for assessing the impact of natural products on the aetiology and metabolic pathophysiology of Type 2 diabetes. Biomed. Pharmacother..

[156] Madhariya R., Dixena B., Ram A., Vyas A., Jain A.K. (2023). Experimental Animal Models: Tools to Investigate Antidiabetic Activity. Curr. Pharm. Des..

[157] Ali Z., Chandrasekera P.C., Pippin J.J. (2018). Animal research for type 2 diabetes mellitus, its limited translation for clinical benefit, and the way forward. Altern. Lab. Anim..

[158] Wojcikowski K., Gobe G. (2014). Animal studies on medicinal herbs: Predictability, dose conversion and potential value. Phytother. Res..

[159] Wang S., Zhu F. (2016). Antidiabetic dietary materials and animal models. Food Res. Int..

[160] Muschietti L., Redko F., Ulloa J. (2020). Adulterants in selected dietary supplements and their detection methods. Drug Test. Anal..

[161] Posadzki P., Watson L., Ernst E. (2013). Contamination and adulteration of herbal medicinal products (HMPs): An overview of systematic reviews. Eur. J. Clin. Pharmacol..

[162] Ng J.Y., Kim M., Suri A. (2022). Exploration of facilitators and barriers to the regulatory frameworks of dietary and herbal supplements: A scoping review. J. Pharm. Policy Pract..

[163] Othman Z.K., Ahmed M.M., Kasimieh O., Musa S.S., Branda F., Cue E.G., Ocampo J.M.A., Lucero Prisno D.E., Vimolmangkang S. (2025). Artificial intelligence for natural product drug discovery and development: Current landscape, applications, and future directions. Intell.-Based Med..

[164] Al Shami R., Mousa W.K. (2026). Mining the Hidden Pharmacopeia: Fungal Endophytes, Natural Products, and the Rise of AI-Driven Drug Discovery. Int. J. Mol. Sci..

